# Home design features post-COVID-19

**DOI:** 10.1186/s44147-022-00142-z

**Published:** 2022-09-30

**Authors:** Nancy H. Alhadedy, Hisham S. Gabr

**Affiliations:** Department of Architecture, Faculty of Engineering, Cairo University, Giza, Egypt

**Keywords:** Residential quality, COVID-19, Lockdown, Housing quality, Well-being, Quality of life, Architectural design, Spaces, Pandemic, Built environment, Post-pandemic, Housing environment

## Abstract

The long stay at home during the COVID-19 pandemic implied that most people had to perform all their daily activities at home. That raised the need for special home features related to the health, safety, and well-being of residents. This study aimed to explore the most essential features of home design during the pandemic and to measure the perception of their importance by Cairenes (Egypt) and construction industry experts for their further implementation in the future home design as part of the buyers’ preferences and house quality features. The study employed an exploratory survey, identifying eight design features, followed by a quantitative questionnaire to measure the importance of each feature among the stakeholders. The results showed that all eight indicators were of a certain degree of importance. It was found that the availability of natural ventilation and natural light were the most essential features, followed by the availability of a private outdoor space, such as a terrace with a good-looking view or a private garden, and the availability of at least one bedroom with an enclosed bathroom for the isolation needs. In contrast, the availability of an extra storage space for food and supplies, as well as the availability of an indoor family entertainment space was reported as the least important.

**Supplementary Information:**

The online version contains supplementary material available at 10.1186/s44147-022-00142-z.

## Introduction

Since COVID-19 (coronavirus disease 2019) was first reported in Wuhan, China, in late December 2019 [1], the virus has rapidly spread to other countries. On January 30, 2020, the World Health Organization (WHO) declared the outbreak of COVID-19, a Public Health Emergency of International Concern (PHEIC) [2]. The COVID-19 virus is extremely fast to disseminate in a variety of different ways, which explains its global expansion. The primary modes of transmission are a direct, close, and indirect contact with an infected person or surfaces [3, 4]. Given the COVID-19 symptoms that are most frequently associated with fever, cough, dyspnea, myalgia, and weariness [5, 6], the WHO urged the governments worldwide to adopt social distancing in order to slow down the rate of transmission and curb the pandemic [7]. Many Egyptians—being no exception—responded to the threat by social distancing; many of them, especially those pursuing vocations linked with a higher social status, voluntarily complied with the working from home requirements [8, 9]. But when the rate of infected cases exponentially increased, in line with the global trend, Egypt’s government ordered lockdown restrictions on all the population who was compelled to stay home except for essential needs. Numerous schools, clubs, and public gardens were forced to close [7]. “Stay Home - Stay Safe!” campaigns to impose social distancing measures on the social networks were launched to increase public awareness of the COVID-19 pandemic. All festivals were halted, and the number of on-site workers in non-vital operations was decreased to encourage public sector employees to work remotely, reducing their interaction and preventing virus spread in the workplaces [7]. As a result, children, the elderly, and a sizable portion of the whole community were forced to remain at home [8]. People found themselves confined for extended periods of time whether voluntarily or by force. Due to such a level of social separation, the pandemic put more social, mental, and psychological challenges on residents’ lives [10], especially that social gatherings and festivals are deeply ingrained in the Egyptian culture [7]. For some strata of the Egyptian population who live in big households and extended families, yet smaller and more cramped dwellings—often with no access to open areas—the measures enforced caused even more frustration [8, 11]. Besides, those measures were not easy to follow, since many residents could neither afford to stay home nor perform their jobs remotely [8]. Hence, a home that had been previously considered a space to relax after a long day of outside activities suddenly became an office, school, restaurant, playground, space for sports, and much more [12]. In addition, a high number of patients infected with COVID-19 had overwhelmed the capacity of hospitals and quarantine institutions; therefore, households operated as places of self-isolation. Finally, the changing lifestyle and unpredictable environment have allowed people to recognize that the only way to cope is by adapting to the challenges of COVID-19 or even other contagious diseases that might emerge in the future [13]. Thus, given the COVID-19 persistence, it is expected that more individuals would work from home even after the quarantine time [14–16]. On the bright side, the pandemic has increased residents’ appreciation of their homes. People have also learnt that working or studying from home may be more efficient and beneficial to the environment, economy, and health. As a result, it becomes obvious that homes must be tweaked to prioritize health and safety measures and to ensure their new role in meeting the new lifestyle requirements. For new homes, measures that align with the new norm should be taken into consideration from the early stage of the design process. The awareness of the designers, realtors, and buyers of these measures is important, especially because the housing industry in Egypt is critical, striving for filling the property shortage of about 3 million units [17], where both the public and private sectors are actively engaged in creating thousands of new units and encouraging the public to purchase new homes [18]. Throughout history, architecture has been always responsive to pandemics and health threats in innovative and adaptive ways [19]. Regarding the stay-home implications, manifold useful—yet insufficient—research in multiple disciplines, including architectural design, environmental psychology, building science, engineering, urban planning, and health care, has been undertaken to determine what modifications are necessary to make homes more adaptable to the new norm [20–23]. The results have shown that housing may be an excellent starting point for design innovation and may serve as a preventive treatment for future pandemics via design. New designs have been proposed to consider dwelling characteristics, such as a housing layout, interior and exterior, building material and furnishing, natural lighting, and indoor air quality, as well as landscape and its value [23]. With regard to energy and water supply, these future designs are envisaged to be self-sufficient and self-contained, minimizing dangers in case of a full shutdown [20, 24]. It is also required to separate heating and ventilation systems in detached residences and multi-story complexes to avoid infectious disease transmission [20]. For interior spaces, the findings show that the design should emphasize transparency, openness to the inside (introverted spaces), the quality of life, natural lighting, and ventilation, as well as the use of plants and natural materials [20–23]. Flexible spaces are to be favored because they may be partitioned into clean and polluted zones, as well as quiet and active zones, thus assisting in the prevention of infections and maintaining daily hygiene [22]. Folding furniture may also be a good option for expanding a space and turning it, if necessary, into multifunctional rooms [20]. House sizes shall be sufficiently large to permit social separation [21, 22], while a home office space for most dwellings becomes mandatory, it can no longer be featured with a little desk, chair, and light tucked away in a dark corner. It should be an enclosed area that is sufficiently far from the living room to minimize noise and interruptions. Besides, it ought to be technologically well-equipped [20, 22]. For bedrooms, at least one separate room that includes a bathroom is required for the purpose of isolation in case anyone in the household gets infected. The indoor air quality and natural light must be sufficient in the space to benefit a patient’s immunity [22, 23]. A second bathroom next to the entryway is necessary for a more regulated disinfecting environment [22]. Terraces and private outdoor areas become important useable spaces for many activities, such as sunbathing, fitness and relaxation, work, and study; hence, a multi-purpose garden for hobbies or even food consumption may become an effective solution to relieving the stress and boredom caused by the isolation and for enhancing immunity [20, 22, 23]. The entryway plays an effective role in separating indoor and outdoor germs when entering a residence. The entryway is usually used for storing shoes, clothes, and outdoor things [22]. Therefore, having a defined entryway or a foyer may prevent future cross-infection. Using touchless and intelligent technologies in architecture and design becomes highly beneficial in controlling disease transmission [20, 22]. Although the recommendations suggested may control the risk of pandemic spread in the home environment and increase the quality of life during quarantines and stay-home periods, they are neither sufficient nor conclusive as the world is still uncertain about the virus and its short-term and long-term effects due to continuous mutations [20].

In addition, relevant studies vary in their location and, consequently, environment, climate, culture, residential and social preferences, political circumstances, and, above all, virus transmission characteristics in such a location [20]. To successfully apply the variety of recommendations, an involved professional should manifest awareness, engagement, and willingness to introduce the above changes in the lifestyle and practices. This also requires the cooperation of all stakeholders involved in the building industry, including designers, realtors, and residents.

These are only some concerns raised by the current pandemic regarding home design features that may facilitate coping with the pandemic-induced stay-at-home situation and may remain a significant component of homes in the future. The exact solutions are uncertain as of the moment. However, they are intimately entwined and are expected to continue to be the subject of extensive research during the global pandemic or subsequent outbreaks with comparable effects [7].

This research aims to answer the following questions: What are the home design features that are proved to be essential during the pandemic and may be considered in the future? How important are they to the Cairenes? How do professionals assess the significance of such features?

To answer these questions, it was important to investigate how Cairo residents coped with the pandemic in their homes, what measures they took, and what design features they needed to ensure safety, health, and well-being. The second phase was to examine the perceived importance of these measures by the residents and specialists involved in the building industry, including designers, engineers, and real-estate professionals.

## Methods

This research aims to explore the required design features of homes that promote safety, health, and well-being during and after the COVID-19 pandemic, as well as to measure the perception of the importance of these features for both home residents and industry professionals in Cairo (Egypt) in order to define them as part of the buyers’ preferences and house quality features for future designs.

**Fig. 1:**
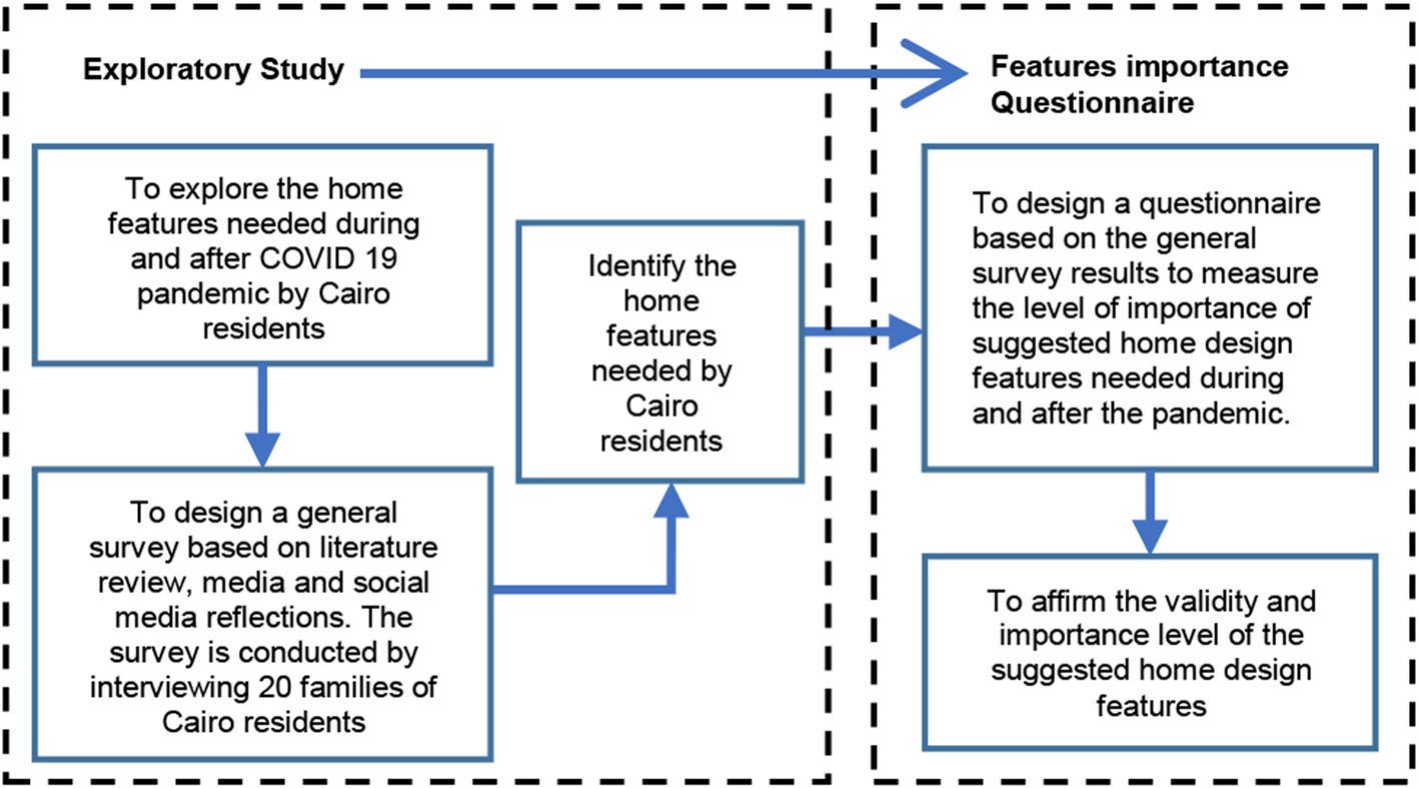
Research methodology diagram. Ref: researchers

To achieve this aim, the researchers conducted an exploratory survey by interviewing twenty families of Cairo residents, to elicit the most appreciated design features during the pandemic followed by a focused quantitative questionnaire to measure the perception of the importance of these features for both home residents and industry professionals in Cairo. The exploratory phase was followed by a quantitative survey to affirm the perceived validity and importance level of the suggested home design features. Figure 1 shows the research methodology diagram.

Because this study was conducted during the pandemic period, social separation was necessary, and hence, all data were collected remotely. The exploratory survey was conducted through phone calls and online meetings using Zoom^TM^. The questionnaire for the quantitative survey was created and disseminated using SurveyMonkey^TM^, an online survey tool. The participants were contacted using social media platforms, such as LinkedIn^TM^, Facebook^TM^, and WhatsApp^TM^. Microsoft Excel^TM^ and SPSS^TM^ were used to perform the statistical analysis of the data.

### Exploratory study

A quantitative questionnaire was used to evaluate the design features of homes needed by Cairenes during the COVID-19 pandemic.

The survey targeted middle and upper class medium size families as they had more control over their lifestyle and a higher ability to alter their living spaces to their changing needs [24]. In contrast, lower socioeconomic layers were found to be less able to adopt and comply with certain non-pharmaceutical interventions during the pandemic [25]. A survey invitation message was prepared and sent via social media channels to a number of people who qualified to participate. The message intent was to obtain approval and pre-agreement for participation in the survey and to request referrals as a snowball sampling approach [26]. Only twenty families out of 25 responded to the request.

The twenty families were interviewed via phone calls and online meetings. The following twelve questions were asked during the interviews: (1) “Where do you live?”, (2) “What is the size of your home?”, (3) “How many family members live in the house?”, (4) “Did any of your family members get COVID-19?”, (5) “How old are they?”, (6) “What were the measures that you took for protection at home?”, (7) “How did the quarantine affect you and your family activities?”, (8) “How did you cope with staying home during the pandemic?”, (9) What were the changes that you made in your home space?”, (10) **“**What were the characteristics and features in your home that helped you the most to cope?”, (11) **“**What are the aspects and features that you wish you had had in your home to raise the quality of life during the quarantine?”, and (12) “What are the changes in your home space that you would rather keep after the pandemic?”.

Families who were interviewed varied in size, family members’ age, areas of residency, and whether they were infected by the virus. All the interviewed families were middle to upper class families who owned their homes and had the freedom and ability to adjust their living spaces according to their needs. Nine families lived in new cities (New Cairo, 6th October, and Sheikh Zayed), three families lived in gated compounds (AlRehab, Madinaty, and Alashgar), and eight families lived in older districts (Nasr City, Heliopolis, and Dokki). Seventeen families lived in apartments and three in villas. The residential space sizes averaged between 110 and 300 m^2^. Ten families had at least one family member infected with COVID-19. The results were found consistent with the literature findings. All the interviewed families agreed that they had to make changes in their spaces, mostly to cope with the work/study from home situation. All families with young kids mentioned that they had to create a special zone for indoor activities for their kids. All the interviewed families mentioned that they had to follow a regular sanitization routine, especially when one of the family members would arrive from outside or when goods and home supplies were brought in from the market. The closest bathroom to the entryway was used for that purpose. All families found that having a private outdoor space, such as a terrace, private garden, or a private roof, was essential for their mental and physical health, especially for the elders and young kids. Families who had one or more of their members infected with the virus emphasized the importance of having at least one master bedroom with an attached bathroom to be used for isolation. It was found that most families wished to have more open flexible spaces to add more activity zones, such as a home office, an indoor play/sports area, an extra room for isolation, and a pantry space to store more food and supplies to reduce the number of visits to the supermarkets.

The results were concluded and summed into two main categories: (i) the health, safety, and security measures and (ii) activities and space functions.

i. Health, safety, and security measures included the need for the following:
  - Well-ventilated and naturally lit indoor spaces
  - A safe storage space to store food and goods
  - At least one room with a bathroom, which could be used as an isolated space for quarantine purposes
  - The entrance of the unit connected to a lobby, but not directly attached to the living room
ii. Activities and space functions included the availability of the following:
  - Enough space to perform family entertainment activities
  - An outdoor private space, such as a terrace/private garden/rooftop
  - Home office or work/study space for work and study needs
  - Flexibility of design and open spaces as a way to change or modify their function

The results about the design features elicited were translated into eight key indicators, which were (1) flexibility/open spaces, (2) natural light and ventilation, (3) indoor entertainment space, (4) food and supply storage, (5) home office, (6) terrace with view/private garden, (7) bedroom with enclosed bathroom, and (8) separated entrance.

Several limitations were identified. Thus, during the exploratory phase, we concentrated on identifying the qualities that would be required in houses during a pandemic, for which only limited information was accessible. Although the characteristics suggested by the participants were often addressed, a more focused investigation was required to validate and measure their significance, using a quantitative questionnaire. Moreover, the exploratory survey focused on more privileged communities, while the challenges of people living in other communities would probably be very different. More studies focusing on the impact across socioeconomic groups, particularly across the labor market should be conducted, aiming to support families equitably across the income spectrum.

### Quantitative survey

Following the collection of generic characteristics through the exploratory study, a quantitative survey was conducted through the perceived features importance questionnaire.

The purpose of the questionnaire was to validate and measure the perceived relevance and importance of the features elicited.

The questionnaire consisted of two main parts with a total of 12 questions in Arabic (the Egyptian dialect). The first part of the questionnaire included personal information about the respondent regarding his/her occupation, place of residency, and resident’s class. All questions were optional. The second part had questions regarding the importance level of eight home design features from the respondent’s point of view.

As mentioned earlier, each feature was assigned to an indicator to be evaluated (see Table 1). The questionnaire asked the following: “From your point of view, to which extent is it important that the following features/elements are to be available at your home in case of outside dangers or quarantines?” The list of features included eight characteristics of the home design: (1) flexibility of design and open spaces as a way to change or modify their function, (2) the availability of natural light and ventilation, (3) the availability of sufficient space to perform family entertainment activities, (4) the availability of a safe storage space for food/supplies, (5) the availability of a home office or work/study space, (6) the availability of a terrace with a good view or a private garden, (7) the availability of at least one room with a bathroom, which could be used as an isolated space for the quarantine purposes, and (8) the entrance of the unit is connected to a lobby and not directly attached to the living room.

**Table 1:** Features of evaluation and their related indicators

	Feature	Indicator
1	Flexibility of design and open spaces as a way to change or modify their function	Flexibility/open spaces
2	The availability of natural light and ventilation	Natural light and ventilation
3	The availability of sufficient space to perform family entertainment activities.	Indoor entertainment space
4	The availability of a safe storage space for food/supplies	Food and supplies storage
5	The availability of home office or work/study space	Home office
6	The availability of a terrace with a good view or a private garden	Terrace with view/private garden
7	The availability of at least one room with a bathroom, which could be used as an isolated space for the quarantine purposes	Bedroom with enclosed bathroom
8	The entrance to the unit is connected to a lobby and not directly attached to the living room.	Separated entrance

Each indicator was weighted from 1 to 9 on a Likert scale [27]. The respondents were asked to weigh the importance of each indicator according to their judgment. Ranging from 1 = “No Importance” to 9 = “Extreme importance.” The used scale was our adaptation of the fundamental scale of absolute numbers borrowed from the work of Saaty [28]. More details about the scale of importance are shown in Table 2.

**Table 2:** Scale of importance

Intensity	Definition	Range
1	No importance	1–1.89
2	Weak or slight importance	1.99–2.78
3	Moderate importance	2.88–3.67
4	Moderate plus	3.77–4.56
5	Strong importance	4.66–5.45
6	Strong plus	5.55–6.34
7	Demonstrated importance	6.44–7.23
8	Very, very strong	7.33–8.12
9	Extreme importance	8.22–9

#### Data collection

The questionnaire targeted Greater Cairo residents, as well as residential construction industry professionals, such as architects, building and construction engineers, interior designers, urban planners, real estate developers, investors, and realtors.

The survey using the questionnaire was conducted during the coronavirus pandemic quarantine, and no physical contact with participants was possible. The questionnaire was built through the SurveyMonkey^TM^ web tool. Using the convenience sampling technique, an invitation of participation message was sent to the prospected audience through email and social media channels such as LinkedIn^TM^, Facebook^TM^, and WhatsApp^TM^ applications.

The message contained a link to the questionnaire, along with a brief introduction to the research and its objectives, an explanation of the questionnaire’s nature, and the anticipated completion time. The letter concluded with a note of appreciation and a call for recommendations as a snowball sampling strategy.

#### Data analysis

The data analysis technique comprised both descriptive and inferential statistical methods. Descriptive statistics was used to measure the central tendency, such as the mean and the dispersion (standard deviation) of the data that was subsequently used to rank the importance level of the home design features during and after COVID-19. Then, Cronbach’s alpha approach was used to measure the reliability of scales. In addition to the mean ranking, it was important to verify if there were any statistically significant differences between the views of respondents of different occupational backgrounds. The participants were grouped into 4 independent groups: (1) the design related professions including architects, urban planners/designers, and interior designers; (2) construction professions including civil engineers, construction engineers, and building contractors; (3) real-estate professions including real-estate developers, real-estate investors, and realtors; and the fourth group included users/residents. The one-way (ANOVA) analysis of variance was performed to determine whether there is a statistically significant difference between the means of the four independent groups.

ANOVA is a parametric test that assumes that the data are normally distributed and with equal variance. The hypotheses used in the ANOVA analysis were as follows:

- H0: The means are equal for each group.
- HA: At least one of the means is different from the others.

**Fig. 2:**
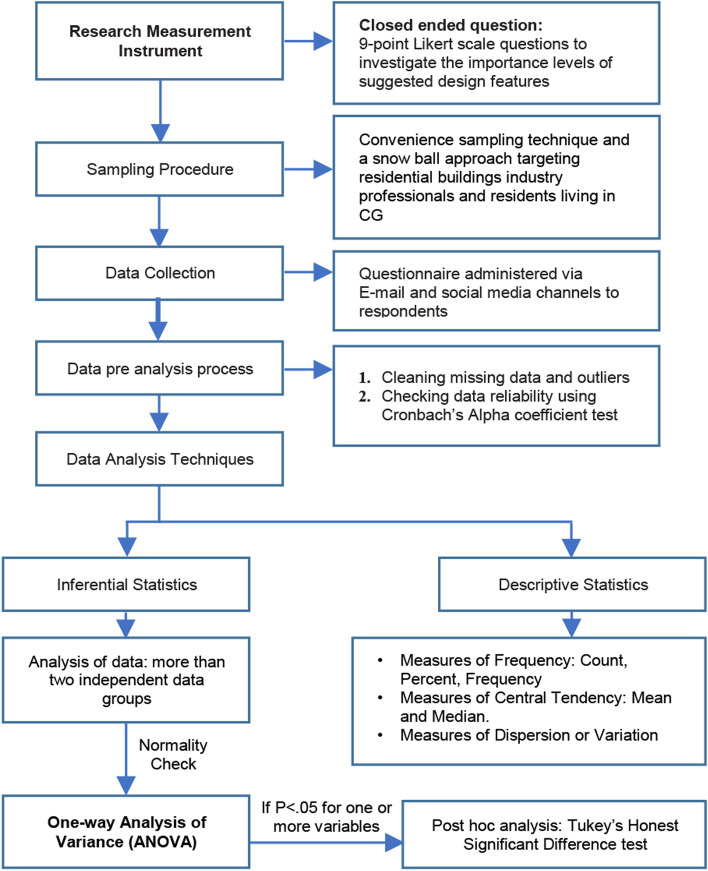
Statistical approach diagram. Ref: researchers

If the *p* value from the ANOVA was less than the significance level *α* = .05, then the null hypothesis would be rejected, indicating that at least one of the group means would be different from the others. If significant results were found, then a post hoc test would be performed—Tukey’s HSD—to find out which specific groups’ means (compared with each other) were different. The test compared all possible pairs of means. Figure 2 shows the statistical approach employed.

## Results

### Demographic data

Among 450 participation attempts to fill the questionnaire, only 439 responses were successfully submitted, and 315 of them completed the questionnaire to the end and matched the audience criteria. All participants lived in Greater Cairo, 266 (84.4%) of the participants were scattered over 18 main areas in Greater Cairo and 49 (15.6%) of them did not declare their residency area, and 90 (28.6%) of them lived in class “A” residencies, 155 (49.2%) in class “B,” 67 (21.2%) in class “C,” and 3 (1%) in class “D.”

One hundred twenty-three of the participants were engineers involved in the building industry, 87 (27.6%) in the design-related professions (architects, interior designers, and urban planners), 36 (11.4%) in construction-related professions (civil, construction engineers, and contractors of different building construction specialties). Thirty-nine (12.4%) of the participants were involved in the field of real estate, and 153 (48.6%) were residents—either owners, tenants, or others. The participants’ details and their distribution are summarized in Table 3.

**Table 3:** Participants’ occupation distribution

Stakeholder	Occupation	Frequency (%)	Subtotal (%)	Total (%)
Design related	Architects	75 (23.8)	87 (27.6)	315 (100)
	Interior designers	4 (1.3)		
	Urban planners	8 (2.5)		
Construction related	Civil/construction engineers	16 (5.1)	36 (11.4)	
	Contractors	20 (6.3)		
Real-estate related	Real-estate developers	19 (6.0)	39 (12.4)	
	Real-estate investors	10 (3.2)		
	Realtors	10 (3.2)		
Users	Residents	153 (48.6)	153 (48.6)	

### Statistical analyses

Statistical analyses were carried out using SPSS Software (IBM^TM^ *SPSS*^TM^). In the beginning, an internal reliability test was performed on the Likert-scaled type questions using Cronbach’s coefficient alpha. The alpha coefficient ranged in value from 0 to 1, where higher values of alpha were more desirable. Cronbach’s alpha coefficient would be interpreted as high reliability (*α* = .9), moderate reliability (*α* = .8), and low reliability (*α* = .7) [29]. The alpha value for the eight home design features was (*α* = .845), indicating that the nine-point Likert scale was reliable. Therefore suitable for more analysis.

Descriptive statistics were used to present the distribution of responses along the home design features showing the number and percentage of responses on each evaluation point from 1 to 9 as shown in Table 4.

**Table 4:** Frequencies of participants’ perceived importance ratings

Indicator	Frequency									
		1	2	3	4	5	6	7	8	9
Flexibility/open spaces	N	1	1	4	7	29	37	78	76	80
	%	0.3	0.3	1.3	2.2	9.2	11.7	24.8	24.1	25.4
Natural light/ventilation	N	0	0	0	0	4	10	36	78	185
	%	0	0	0	0	1.3	3.2	11.4	24.8	58.7
Indoor entertainment space	N	2	2	5	13	32	42	84	56	79
	%	0.6	0.6	1.6	4.1	10.2	13.3	26.7	17.8	25.1
Food/supplies storage	N	3	3	12	12	43	45	90	48	59
	%	1.0	1.0	3.8	3.8	13.7	14.3	28.6	15.2	18.7
Home office	N	3	2	2	5	31	33	78	76	85
	%	1.0	0.6	0.6	1.6	9.8	10.5	24.8	24.1	27.0
Terrace/garden	N	0	2	2	3	17	23	42	67	159
	%	0	0.6	0.6	1.0	5.4	7.3	13.3	21.3	50.5
Bedroom with bathroom	N	2	1	6	5	18	22	55	64	142
	%	0.6	0.3	1.9	1.6	5.7	7.0	17.5	20.3	45.1
Separated entrance	N	5	2	4	16	37	34	58	74	85
	%	1.6	0.6	1.3	5.1	11.7	10.8	18.4	23.5	27.0

*N* number of participants; total number of participants=315

**Fig. 3:**
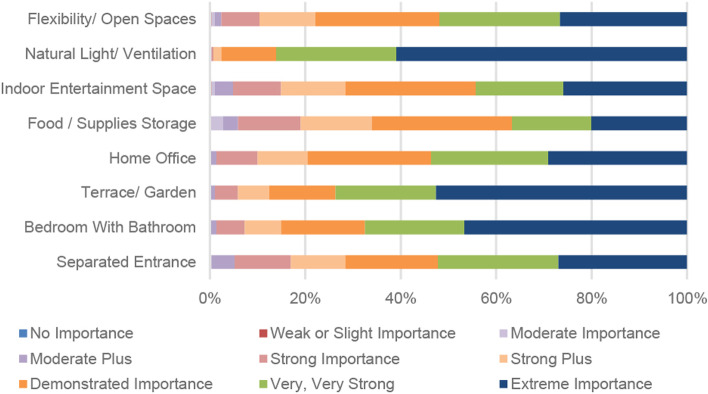
Participants’ perceptions of the importance of the suggested home design indicators during and post-COVID-19. Ref: researchers

The results showed that participants found all the features of significant importance; 95% of them rated all the features’ importance level as of “Strong Importance”, “Strong Plus”, “Very Strong”, “Very, Very Strong,” or “Extreme Importance.” Figure 3 shows the participants’ perceptions of the importance of the suggested home design indicators during and after COVID-19.

**Fig. 4:**
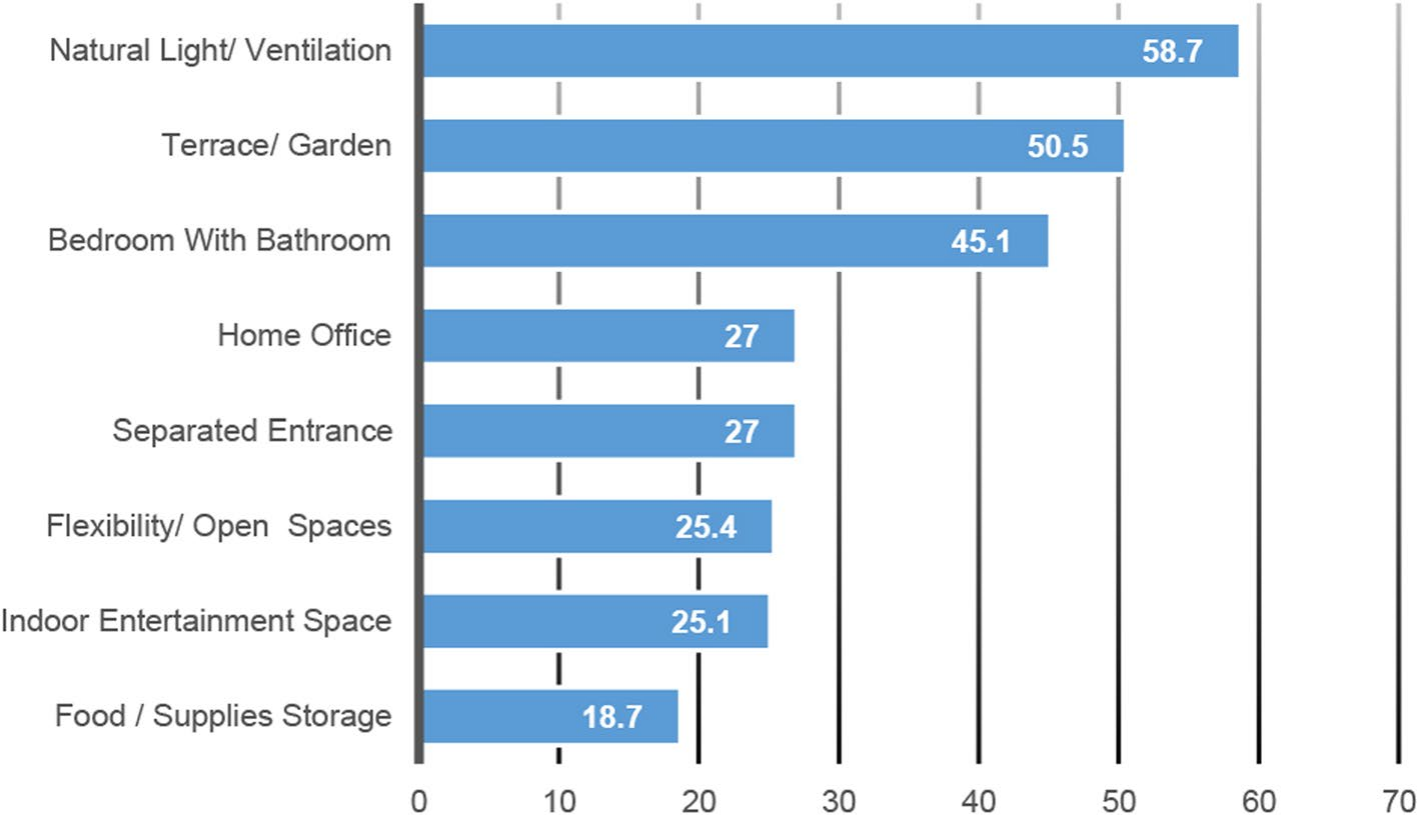
Percentage of participants’ ratings of indicators being of “Extreme Importance” ranked by means. Ref: researchers

The top-rated feature indicators perceived as of “Extreme Importance” were related to the following indicators: natural light and ventilation (58.7%), terrace with view/private garden (50.5%), and bedroom with enclosed bathroom (45.1%). Features least often rated as being of “Extreme Importance” comprised the indicators of home office (27%), separated entrance (27%), flexibility/open spaces (25.4%), indoor entertainment space (25.1%), and food and supply storage (18.7%). Figure 4 shows the participants’ ratings of indicators being of “Extreme Importance” ranked by the means.

More descriptive analysis was conducted to describe the mean, median, standard deviation, the minimum and maximum values, skewness, and kurtosis values as shown in Table 5. The results described the mean value of responses which was the arithmetic mean across the observations. In this sample, all mean values were above 6.5 which indicates that all design features presented a distinctive degree of importance. The median—the midpoint of the distribution in this sample—was 9 for the natural light/ventilation and terrace/garden indicators, which was the highest value on the scale; the median for home office, bedroom with enclosed bathroom, and separated entrance was 8, while the median for flexibility/open spaces, indoor entertainment space, and food/supply storage was 7. The high value of the median for all the indicators confirms that the participants perceived all the features as those of high importance. The standard deviation of the distribution measured the spread of a set of observations. For this sample, the standard deviation was narrow, which indicated that the data were clustered around the mean. The minimum showed the smallest value of the variable; in this sample, the minimum value for all the indicators was 1, which was the lowest value on the scale, except for the indicator of natural light/ventilation whose minimum value was 3, and the terrace/garden indicator, which was 2. The maximum, which was the largest value of the variable, scored 9 for all the design indicators. Skewness measured the degree and direction of asymmetry, while kurtosis measured the heaviness of the tails of the distribution, describing the shape of the data and its normality. The data are considered to be normal if skewness is between −2 and +2 and kurtosis is between −7 and +7 [30–32]. For this sample, the skewness and kurtosis values fell in the range for all the design indicators, showing that the sample was normally distributed.

**Table 5:** Descriptive statistics

Indicators	Mean	Median	Std.	Skewness	Kurtosis	Min	Max
Flexibility/open spaces	7.30	7.00	1.508	−0.922	0.861	1	9
Natural light/ventilation	8.36	9.00	0.949	−1.804	4.168	3	9
Indoor entertainment space	7.09	7.00	1.654	−0.839	0.625	1	9
Food/supplies storage	6.74	7.00	1.751	−0.732	0.377	1	9
Home office	7.32	8.00	1.572	−1.184	1.948	1	9
Terrace/garden	7.96	9.00	1.400	−1.540	2.308	2	9
Bedroom with bathroom	7.74	8.00	1.586	−1.551	2.524	1	9
Separated entrance	7.10	8.00	1.812	−1.028	0.834	1	9

*N* number of participants, *Mean* the average of the sample, *Median* the middle value, *Std.* standard deviation, *Skewness* the measure of the asymmetry of the distribution, *Kurtosis* the sharpness of the peak of a frequency-distribution curve, *Min* the minimum value in the dataset, and *Max* the maximum value in the dataset

To verify if there were any significant differences between respondents’ views of different occupational backgrounds, the one-way ANOVA analysis was conducted.

For that purpose, the participants were grouped into 4 independent groups per occupation. The first group was the design-related professions, including architects, urban planners/designers, and interior designers; the second group was the construction-related professions, such as civil engineers, construction engineers, and building contractors; the third group was related to real-estate professions including real-estate developers, real-estate investors, and realtors; the fourth group included residents of Greater Cairo.

Then, the one-way ANOVA analysis was performed using SPSS to determine the distribution of responses for each occupation, as well as to conclude whether or not there is a statistically significant difference between the means of the four independent groups. Table 6 shows the results values of the ANOVA test.

**Table 6:** One-way ANOVA results

Indicator	Occupation	***N***	Mean	Std.	SE	Min.	Max.	***F***	Sig
Flexibility/open spaces	Design	87	7.26	1.49	0.160	3	9	.837	.474
	Construction	35	6.94	1.88	0.317	1	9		
	Real estate	39	7.36	1.42	0.228	4	9		
	Residents	152	7.38	1.45	0.117	3	9		
	Total	313	7.30	1.51	0.085	1	9		
Natural light/ventilation	Design	86	8.38	0.91	0.098	5	9	.911	.436
	Construction	35	8.43	0.92	0.155	6	9		
	Real estate	39	8.15	1.11	0.178	5	9		
	Residents	153	8.41	0.83	0.067	5	9		
	Total	313	8.37	0.90	0.051	5	9		
Indoor entertainment space	Design	87	7.16	1.66	0.178	1	9	3.622	.013
	Construction	36	6.75	2.05	0.341	2	9		
	Real estate	39	6.41	1.83	0.293	1	9		
	Residents	153	7.29	1.45	0.117	2	9		
	Total	315	7.09	1.65	0.093	1	9		
Food/supplies storage	Design	87	6.77	1.52	0.162	3	9	1.551	.201
	Construction	36	6.36	2.07	0.345	1	9		
	Real estate	39	6.38	1.82	0.291	1	9		
	Residents	153	6.90	1.77	0.143	1	9		
	Total	315	6.74	1.75	0.099	1	9		
Home office	Design	87	7.30	1.42	0.152	3	9	3.381	.019
	Construction	36	6.69	1.82	0.303	1	9		
	Real estate	39	7.05	1.62	0.260	3	9		
	Residents	153	7.54	1.55	0.125	1	9		
	Total	315	7.32	1.57	0.089	1	9		
Terrace/garden	Design	87	7.85	1.39	0.149	3	9	1.157	.326
	Construction	36	7.69	1.64	0.273	3	9		
	Real estate	39	7.87	1.45	0.233	4	9		
	Residents	153	8.10	1.33	0.107	2	9		
	Total	315	7.96	1.40	0.079	2	9		
Bedroom with bathroom	Design	87	7.84	1.42	0.152	3	9	.757	.519
	Construction	36	7.42	1.65	0.274	3	9		
	Real estate	39	7.62	1.63	0.261	3	9		
	Residents	153	7.80	1.65	0.134	1	9		
	Total	315	7.74	1.59	0.089	1	9		
Separated entrance	Design	87	7.60	1.66	0.178	1	9	3.133	.026
	Construction	36	6.94	1.71	0.284	3	9		
	Real estate	39	6.79	1.91	0.306	2	9		
	Residents	153	6.93	1.86	0.150	1	9		
	Total	315	7.10	1.81	0.102	1	9		

*N* number of participants, *Mean* the average of the sample, *Std.* standard deviation, *SE* standard deviation error, *Min* the minimum value in the dataset, *Max*. the maximum value in the dataset, *F* the *F* value; the ratio of mean squares, *Sig.* the *P* value/value of significance <0.05

**Fig. 5:**
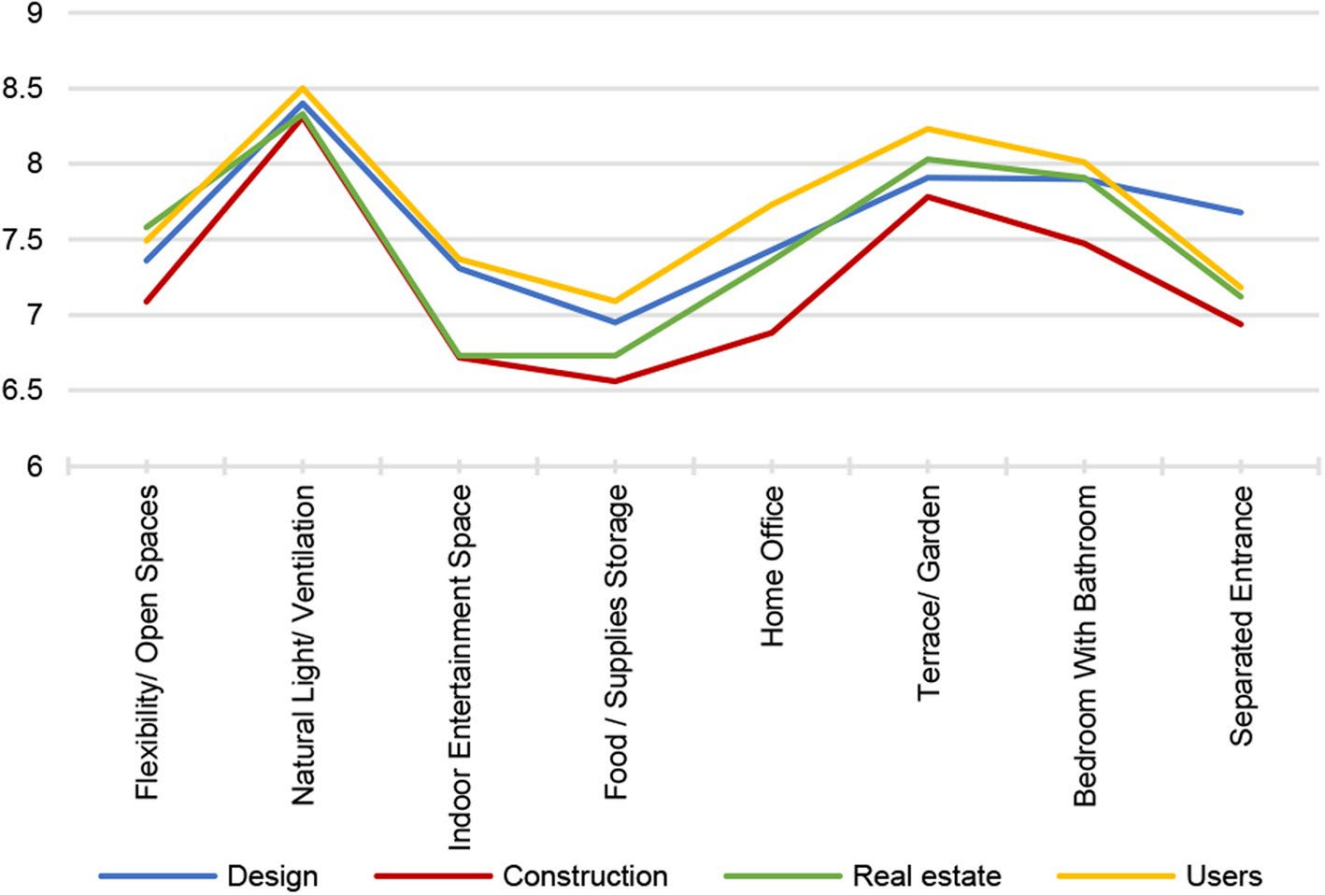
Perceived importance levels per occupation. Ref: researchers

When comparing the means between the groups, we found that all professionals reported that the natural light and ventilation indicator was of the highest importance, followed by the terrace/garden indicator. Unlike all other groups, the designers perceived the separate entry as critical. The users/residents also believed that bedroom with an enclosed bathroom was of high importance, sharing the same views with the designers and real-estate professionals. Additionally, all groups in the sample ranked food and supply storage as the least important feature. The real-estate and construction engineers also valued the indoor entertainment space less than the designers and residents. Conversely, the construction engineers perceived the home office as a less important indicator than other professions. To illustrate the perceived importance of each occupation, Fig. 5 was generated by combining the importance of rating means.

To determine if the differences in views between the groups were statistically significant, the *p* value was compared. If the *p* value from the ANOVA was less than the significance level *α* = .05, then the null hypothesis would be rejected, which would indicate that at least one of the group means was different from the others.

By comparing the observed significance level (*p* value) to the alpha error level (.05), it was found that the significance level was larger than the alpha error level among all groups, for all the indicators, except for the indoor entertainment space (*p* = .013), home office (*p* = .019), and separated entrance (*p* = .026). The results above implied that only perceptions about the importance of those three design indicators showed statistically significant group differences.

To find out which specific groups’ means (compared with each other) were significantly different, a post hoc Tukey’s HSD test was conducted as shown in Table 7. The results revealed that the residents’ perception of the importance of the indoor entertainment space design indicator was significantly higher than that reported by the real-estate-related professions (*p* = .15). As for the home office design indicator, the group of construction-related professionals and residents had a statistically significant difference in its perception, the users/residents appeared to perceive the home office indicator as significantly more important than the construction related professionals (*p* = .018).

**Table 7:** Post hoc Tukey’s HSD test

Dependent variable	I-Course	J-Course	Mean difference (I-J)	SE	Sig.	95% Confidence interval	
						Lower bound	Upper bound
Entertainment space	Residents	Design	0.133	0.219	0.930	−0.43	0.70
		Construction	0.544	0.303	0.276	−0.24	1.33
		Real estate	0.884^a^	0.293	0.015	0.13	1.64
Home office	Residents	Design	0.244	0.209	0.648	−0.30	0.78
		Construction	0.848^a^	0.288	0.018	0.10	1.59
		Real estate	0.491	0.279	0.294	−0.23	1.21
Separate entrance	Residents	Design	−0.663^a^	0.241	0.032	−1.29	−0.04
		Construction	−0.010	0.332	1.000	−0.87	0.85
		Real estate	0.140	0.322	0.973	−0.69	0.97

Mean difference (I-J): Difference in the average value between course I (residents) and course J (other occupations), *SE* standard deviation error, *Sig. P* value/value of significance <0.05

95% confidence interval: 95% probability that the population parameter will fall between the lower and upper bound

^a^The mean difference is significant at the 0.05 level

Besides, it appeared that the design-related professionals perceived the importance of separated entrance as significantly higher than the residents (*p* = .032).

## Discussion

In general, the findings of this research align with the literature review explored. Although it is early to be confident about the outcomes as we are still examining the effects and aftermath of COVID-19, the research has attempted to reveal some home space features that may facilitate residents’ adaptation to challenging conditions. These features were found to be associated with the overall quality of housing, contributing to the enhancement of the housing environment, residents’ overall well-being, and life quality [33]. Therefore, including these considerations in future designs would potentially contribute to sustainable development. According to the research results, the most significant feature to be considered is the availability of natural light and ventilation. In general, studies have proved that natural light and ventilation in households are the key elements for maintaining residents’ health and well-being [34]. Their impact on promoting health and resisting infections has been well recognized. Yet, the implementation of this feature in the design has faced certain challenges. Thus, prior to the COVID-19 outbreak, the world was already concerned with the energy consumption levels in the households and other negative effects on the environment due to the excessive use of mechanical ventilation systems [14]. Promoting natural lighting and ventilation solutions for future home designs will support residents’ health and well-being and will positively contribute to preserving the environment and sustainable development.

Another important design feature revealed in this research is a home office space. Our findings have confirmed the significance of having a home office space as a desirable home design feature. Working and studying from home is expected to become the “new normal” in people’s lifestyle. An interest in the effects of working from home is not new. Thus, various research before COVID-19 had reported a positive relationship between working from home (WFH) and organizational outcomes of productivity, retention, turnover intent, commitment, and performance [35–37]. The onset and rapid development of the COVID-19 pandemic in early 2020 fueled the interest in WFH as millions of people were forced to socially isolate themselves to control the spread of the virus that has allowed for an “enforced experiment,” offering a significant learning opportunity for organizations to evaluate what “works” and what does not, and in what ways WFH could be best managed to benefit both workers and organizations [38]. Besides, WFH has been found to promote sustainable cities, especially in terms of urban environmental management and spatial planning. In line with facilitating urban sustainability, WFH has shown a positive impact on reducing traffic congestion, air pollution, and the need for space for offices, which ensures a reduction in the burden on cities in the future by reducing the accumulation of activity in the city center [39]. Hence, it is not surprising that the users/residents in our study perceived the home office space as essential. Given the relative neglect of this feature by the construction-related professionals revealed in this study, we believe it is crucial to communicate our findings to the target professionals. We recommend that the home office space is technologically well-equipped and planned in an enclosed area, sufficiently far from the living room to minimize noise and interruptions [20, 22].

Flexible open spaces have also been favored under pandemic conditions because they may be partitioned into clean and polluted zones, as well as quiet and active zones, which serve as a barrier to infections and facilitate daily hygiene [22]. Besides, flexibility in design enables residents to personalize, adapt, and adjust the space with the interior environment to meet their changing demands, hence giving a larger range of options than moving to a new location. It can provide a more comfortable household environment in every way, benefiting the resident’s physical, mental, and socio-emotional health, thus allowing for social and physical change in housing seems self-evidently rational [40].

The availability of a terrace with a nice view, or a private garden, food and supplies storage, bedroom with an enclosed bathroom, indoor entertainment space, and separated entrance were found to be significant for the residents’ well-being during the pandemic. They will continue to positively affect the quality of their lives inside homes in the future, whether there is an outside threat or not.

When comparing the ratings by occupation, it was found that all occupations shared almost the same perception regarding the order of features’ importance, with differences in the level of significance. These results highlight the need for some home features to cope with the stay-home situation during the pandemic and after. They are meant to be part of future home designs that promote health, safety, and well-being. It is recommended that designers and design firms optimize their design strategies further according to the new residential needs and take the design of healthy and smart housing as the added value to future residential building products. The new idea of healthy housing in the post-pandemic age must be included in housing development regulations. Hence, the concept of healthy housing should be incorporated into housing design, construction, and operation to enhance the integrity of the building life cycle. Besides, relevant government departments and agencies should proactively create new residential building design standards and norms, as well as build a system of cross-domain practice, including disease prevention and control in the design of residential communities.

## Conclusions

COVID-19 has affected people’s lifestyles in a way of no return; it forced them to rely on their homes to adapt to the new conditions. New demands for homes have emerged, and they should be considered in the upcoming residential project designs. This study has explored some of these demands regarding health, safety, security, and daily activities’ spatial needs, in Cairo, Egypt, residents’ and industry professionals’ point of view. They were expressed through eight indicators, whose importance perceived by the involved stakeholders was evaluated. Although there was a variance in the perception of importance among different professionals, all indicators showed a degree of significance. The total average of the results allowed us to elicit the key features that have been rated as the most significant for both the residents and professionals.

The top-rated features of an extreme importance were good environmental conditions inside homes, such as proper natural ventilation and natural light, having a private access to the outdoors from homes in the form of a private terrace with a good view or a private garden, and having at least one bedroom with an enclosed bathroom that could be used as a self-isolation space in case of disease infection. Other features that were perceived as significantly important, yet not of an extreme importance, included the availability of the home office and the unit entrance connected to a lobby, in addition to flexible open spaces that might be altered to meet the changing needs, and family entertainment space. The least important feature was having a food and supply storage.

As emphasized earlier in this paper, all groups of professionals shared almost the same perception regarding the ranking of home design features, yet showed a statistically significant difference in the perceived level of their significance. However, there was a significant difference between the views of users/residents and professionals in respect of which feature should be considered as extremely important. This implicates that the building industry professionals need to align their views to the residents’ needs. It is recommended that housing developers pay greater attention to the new needs of residents and establish marketing approaches based on actual economic situations. Furthermore, designers should re-adapt to the application capabilities of new requirements in response to changing user demands in the post-pandemic age. This research performed a preliminary investigation based on the residential requirements of urban Egyptians in Greater Cairo during the pandemic.

Although the findings of this research may give valuable insights about the home design features of other countries and regions with comparable characteristics, this study has certain limitations. The first constraint is related to the sample size and residential class of the participant: only the research sample of Greater Cairo was addressed. Second, the research results may be generalized with some caution due to the sample’s distinct climatic and economic situations and since individuals may have varied preferences when selecting home design features. In the future, it is suggested that additional in-depth study is undertaken in other regions. Third, in terms of data collection, this study is limited by the fact that only 20 families participated in the qualitative survey, but the results are deemed sufficient for providing an overview of the to-be-studied home characteristics; besides, their significance was confirmed by the 315 participants of experts and residents of the quantitative questionnaire that followed. Nonetheless, more data collected during the qualitative survey phase would have surely enhanced the research. Fourth, the quantitative study targeted middle and upper class medium size families, as they had more control over their lifestyle and better ability to alter their living spaces to their changing needs. The challenges of people living in less privileged communities would probably be very different. More studies focusing on home needs during pandemics across socioeconomic groups, particularly across the labor market ones, should be conducted, aiming to support families equitably across the income spectra.

Fifth, this research focused on some of the indoor residential features while it did not look at other aspects of dwellings, such as elevators, stairways, and common rooms. It did not focus on housing for accessibility, cost, or people with disabilities either. Future research might look at these concerns and their impact on the residential quality from a post-COVID-19 viewpoint.

Although it is too early to draw definitive conclusions because we are still studying the virus effects and aftereffects, transdisciplinary collaboration across diverse segments of society, professions, and academics in order to solve complex societal concerns caused by the pandemic is crucial. In this regard, this study has contributed to the production of knowledge about post-pandemic architecture and urbanism. In particular, the significance of this research is that it has identified certain characteristics of the home design that both Cairenes and professionals perceived as essential for their homes during and after the pandemic. The features identified are found to be linked to the overall housing quality potentially capable of improving the living environment, residents’ general wellness, and quality of life. We envisage a number of research perspectives, including those related to (1) the cost-optimization of further design solutions in order to give premium assessments to building developers and occupants and (2) alignment of the new design requirements across all the stakeholders in the post-pandemic period.

## Supplementary Information

Additional file 1.

Additional file 2.

Additional file 3.

## Data Availability

The SPSS dataset for the questionnaire analysis was submitted. Additional data that support the findings of this study (original answers to interview questions) are available from the corresponding author upon reasonable request.

## Abbreviations

COVID-19: Coronavirus disease of 2019
WHO: World Health Organization
PHEIC: Public Health Emergency Of International Concern
