# Supplementary material for: Home design features post-COVID-19
Source: J. Eng. Appl. Sci. 2022 Sep 30;69(1):87. doi: 10.1186/s44147-022-00142-z (PMC9523634; doi:10.1186/s44147-022-00142-z)
Supplement: Supplementary file 2 — Additional file 2. [file 44147_2022_142_MOESM2_ESM.docx]

**Frequencies**

| **Notes** | | |
| --- | --- | --- |
| Output Created | | 10-JUL-2022 19:44:41 |
| Comments | |  |
| Input | Data | C:\Users\Nancy\Documents\Phd\Springer Nature Journal Paper phase 2\Latest-107DATABASE SPSS-Home Features Post COVID 19.sav |
|  | Active Dataset | DataSet3 |
|  | Filter | <none> |
|  | Weight | <none> |
|  | Split File | <none> |
|  | N of Rows in Working Data File | 315 |
| Missing Value Handling | Definition of Missing | User-defined missing values are treated as missing. |
|  | Cases Used | Statistics are based on all cases with valid data. |
| Syntax | | FREQUENCIES VARIABLES=Flixibility NaturalLight EnteSpace Storage Homeoffice Terrace Bedroom Entrance /STATISTICS=STDDEV MINIMUM MAXIMUM MEAN MEDIAN SKEWNESS SESKEW KURTOSIS SEKURT /ORDER=ANALYSIS. |
| Resources | Processor Time | 00:00:00.00 |
|  | Elapsed Time | 00:00:00.00 |

[DataSet3] C:\Users\Nancy\Documents\Phd\Springer Nature Journal Paper phase 2\Latest-107DATABASE SPSS-Home Features Post COVID 19.sav

| **Statistics** | | | | | | | | | |
| --- | --- | --- | --- | --- | --- | --- | --- | --- | --- |
|  | | Flixibility | NaturalLight | EnteSpace | Storage | Homeoffice | Terrace | Bedroom | Entrance |
| N | Valid | 313 | 314 | 315 | 315 | 315 | 315 | 315 | 315 |
|  | Missing | 2 | 1 | 0 | 0 | 0 | 0 | 0 | 0 |
| Mean | | 7.30 | 8.36 | 7.09 | 6.74 | 7.32 | 7.96 | 7.74 | 7.10 |
| Median | | 7.00 | 9.00 | 7.00 | 7.00 | 8.00 | 9.00 | 8.00 | 8.00 |
| Std. Deviation | | 1.508 | .949 | 1.654 | 1.751 | 1.572 | 1.400 | 1.586 | 1.812 |
| Skewness | | -.922 | -1.804 | -.839 | -.732 | -1.184 | -1.540 | -1.551 | -1.028 |
| Std. Error of Skewness | | .138 | .138 | .137 | .137 | .137 | .137 | .137 | .137 |
| Kurtosis | | .861 | 4.168 | .625 | .377 | 1.948 | 2.308 | 2.524 | .834 |
| Std. Error of Kurtosis | | .275 | .274 | .274 | .274 | .274 | .274 | .274 | .274 |
| Minimum | | 1 | 3 | 1 | 1 | 1 | 2 | 1 | 1 |
| Maximum | | 9 | 9 | 9 | 9 | 9 | 9 | 9 | 9 |

**Frequency Table**

| **Flixibility** | | | | | |
| --- | --- | --- | --- | --- | --- |
|  | | Frequency | Percent | Valid Percent | Cumulative Percent |
| Valid | 1 | 1 | .3 | .3 | .3 |
|  | 2 | 1 | .3 | .3 | .6 |
|  | 3 | 4 | 1.3 | 1.3 | 1.9 |
|  | 4 | 7 | 2.2 | 2.2 | 4.2 |
|  | 5 | 29 | 9.2 | 9.3 | 13.4 |
|  | 6 | 37 | 11.7 | 11.8 | 25.2 |
|  | 7 | 78 | 24.8 | 24.9 | 50.2 |
|  | 8 | 76 | 24.1 | 24.3 | 74.4 |
|  | 9 | 80 | 25.4 | 25.6 | 100.0 |
|  | Total | 313 | 99.4 | 100.0 |  |
| Missing | System | 2 | .6 |  |  |
| Total | | 315 | 100.0 |  |  |

| **NaturalLight** | | | | | |
| --- | --- | --- | --- | --- | --- |
|  | | Frequency | Percent | Valid Percent | Cumulative Percent |
| Valid | 3 | 1 | .3 | .3 | .3 |
|  | 5 | 4 | 1.3 | 1.3 | 1.6 |
|  | 6 | 10 | 3.2 | 3.2 | 4.8 |
|  | 7 | 36 | 11.4 | 11.5 | 16.2 |
|  | 8 | 78 | 24.8 | 24.8 | 41.1 |
|  | 9 | 185 | 58.7 | 58.9 | 100.0 |
|  | Total | 314 | 99.7 | 100.0 |  |
| Missing | System | 1 | .3 |  |  |
| Total | | 315 | 100.0 |  |  |

| **EnteSpace** | | | | | |
| --- | --- | --- | --- | --- | --- |
|  | | Frequency | Percent | Valid Percent | Cumulative Percent |
| Valid | 1 | 2 | .6 | .6 | .6 |
|  | 2 | 2 | .6 | .6 | 1.3 |
|  | 3 | 5 | 1.6 | 1.6 | 2.9 |
|  | 4 | 13 | 4.1 | 4.1 | 7.0 |
|  | 5 | 32 | 10.2 | 10.2 | 17.1 |
|  | 6 | 42 | 13.3 | 13.3 | 30.5 |
|  | 7 | 84 | 26.7 | 26.7 | 57.1 |
|  | 8 | 56 | 17.8 | 17.8 | 74.9 |
|  | 9 | 79 | 25.1 | 25.1 | 100.0 |
|  | Total | 315 | 100.0 | 100.0 |  |

| **Storage** | | | | | |
| --- | --- | --- | --- | --- | --- |
|  | | Frequency | Percent | Valid Percent | Cumulative Percent |
| Valid | 1 | 3 | 1.0 | 1.0 | 1.0 |
|  | 2 | 3 | 1.0 | 1.0 | 1.9 |
|  | 3 | 12 | 3.8 | 3.8 | 5.7 |
|  | 4 | 12 | 3.8 | 3.8 | 9.5 |
|  | 5 | 43 | 13.7 | 13.7 | 23.2 |
|  | 6 | 45 | 14.3 | 14.3 | 37.5 |
|  | 7 | 90 | 28.6 | 28.6 | 66.0 |
|  | 8 | 48 | 15.2 | 15.2 | 81.3 |
|  | 9 | 59 | 18.7 | 18.7 | 100.0 |
|  | Total | 315 | 100.0 | 100.0 |  |

| **Homeoffice** | | | | | |
| --- | --- | --- | --- | --- | --- |
|  | | Frequency | Percent | Valid Percent | Cumulative Percent |
| Valid | 1 | 3 | 1.0 | 1.0 | 1.0 |
|  | 2 | 2 | .6 | .6 | 1.6 |
|  | 3 | 2 | .6 | .6 | 2.2 |
|  | 4 | 5 | 1.6 | 1.6 | 3.8 |
|  | 5 | 31 | 9.8 | 9.8 | 13.7 |
|  | 6 | 33 | 10.5 | 10.5 | 24.1 |
|  | 7 | 78 | 24.8 | 24.8 | 48.9 |
|  | 8 | 76 | 24.1 | 24.1 | 73.0 |
|  | 9 | 85 | 27.0 | 27.0 | 100.0 |
|  | Total | 315 | 100.0 | 100.0 |  |

| **Terrace** | | | | | |
| --- | --- | --- | --- | --- | --- |
|  | | Frequency | Percent | Valid Percent | Cumulative Percent |
| Valid | 2 | 2 | .6 | .6 | .6 |
|  | 3 | 2 | .6 | .6 | 1.3 |
|  | 4 | 3 | 1.0 | 1.0 | 2.2 |
|  | 5 | 17 | 5.4 | 5.4 | 7.6 |
|  | 6 | 23 | 7.3 | 7.3 | 14.9 |
|  | 7 | 42 | 13.3 | 13.3 | 28.3 |
|  | 8 | 67 | 21.3 | 21.3 | 49.5 |
|  | 9 | 159 | 50.5 | 50.5 | 100.0 |
|  | Total | 315 | 100.0 | 100.0 |  |

| **Bedroom** | | | | | |
| --- | --- | --- | --- | --- | --- |
|  | | Frequency | Percent | Valid Percent | Cumulative Percent |
| Valid | 1 | 2 | .6 | .6 | .6 |
|  | 2 | 1 | .3 | .3 | 1.0 |
|  | 3 | 6 | 1.9 | 1.9 | 2.9 |
|  | 4 | 5 | 1.6 | 1.6 | 4.4 |
|  | 5 | 18 | 5.7 | 5.7 | 10.2 |
|  | 6 | 22 | 7.0 | 7.0 | 17.1 |
|  | 7 | 55 | 17.5 | 17.5 | 34.6 |
|  | 8 | 64 | 20.3 | 20.3 | 54.9 |
|  | 9 | 142 | 45.1 | 45.1 | 100.0 |
|  | Total | 315 | 100.0 | 100.0 |  |

| **Entrance** | | | | | |
| --- | --- | --- | --- | --- | --- |
|  | | Frequency | Percent | Valid Percent | Cumulative Percent |
| Valid | 1 | 5 | 1.6 | 1.6 | 1.6 |
|  | 2 | 2 | .6 | .6 | 2.2 |
|  | 3 | 4 | 1.3 | 1.3 | 3.5 |
|  | 4 | 16 | 5.1 | 5.1 | 8.6 |
|  | 5 | 37 | 11.7 | 11.7 | 20.3 |
|  | 6 | 34 | 10.8 | 10.8 | 31.1 |
|  | 7 | 58 | 18.4 | 18.4 | 49.5 |
|  | 8 | 74 | 23.5 | 23.5 | 73.0 |
|  | 9 | 85 | 27.0 | 27.0 | 100.0 |
|  | Total | 315 | 100.0 | 100.0 |  |

**Explore**

| **Notes** | | |
| --- | --- | --- |
| Output Created | | 10-JUL-2022 20:00:01 |
| Comments | |  |
| Input | Data | C:\Users\Nancy\Documents\Phd\Springer Nature Journal Paper phase 2\Latest-107DATABASE SPSS-Home Features Post COVID 19.sav |
|  | Active Dataset | DataSet3 |
|  | Filter | <none> |
|  | Weight | <none> |
|  | Split File | <none> |
|  | N of Rows in Working Data File | 315 |
| Missing Value Handling | Definition of Missing | User-defined missing values for dependent variables are treated as missing. |
|  | Cases Used | Statistics are based on cases with no missing values for any dependent variable or factor used. |
| Syntax | | EXAMINE VARIABLES=Groups Flixibility NaturalLight EnteSpace Storage Homeoffice Terrace Bedroom Entrance /PLOT BOXPLOT STEMLEAF /COMPARE GROUPS /STATISTICS DESCRIPTIVES EXTREME /CINTERVAL 95 /MISSING LISTWISE /NOTOTAL. |
| Resources | Processor Time | 00:00:02.48 |
|  | Elapsed Time | 00:00:01.29 |

| **Case Processing Summary** | | | | | | |
| --- | --- | --- | --- | --- | --- | --- |
|  | Cases | | | | | |
|  | Valid | | Missing | | Total | |
|  | N | Percent | N | Percent | N | Percent |
| Groups | 312 | 99.0% | 3 | 1.0% | 315 | 100.0% |
| Flixibility | 312 | 99.0% | 3 | 1.0% | 315 | 100.0% |
| NaturalLight | 312 | 99.0% | 3 | 1.0% | 315 | 100.0% |
| EnteSpace | 312 | 99.0% | 3 | 1.0% | 315 | 100.0% |
| Storage | 312 | 99.0% | 3 | 1.0% | 315 | 100.0% |
| Homeoffice | 312 | 99.0% | 3 | 1.0% | 315 | 100.0% |
| Terrace | 312 | 99.0% | 3 | 1.0% | 315 | 100.0% |
| Bedroom | 312 | 99.0% | 3 | 1.0% | 315 | 100.0% |
| Entrance | 312 | 99.0% | 3 | 1.0% | 315 | 100.0% |

| **Descriptives** | | | | |
| --- | --- | --- | --- | --- |
|  | | | Statistic | Std. Error |
| Groups | Mean | | 2.82 | .073 |
|  | 95% Confidence Interval for Mean | Lower Bound | 2.68 |  |
|  |  | Upper Bound | 2.97 |  |
|  | 5% Trimmed Mean | | 2.86 |  |
|  | Median | | 3.00 |  |
|  | Variance | | 1.676 |  |
|  | Std. Deviation | | 1.295 |  |
|  | Minimum | | 1 |  |
|  | Maximum | | 4 |  |
|  | Range | | 3 |  |
|  | Interquartile Range | | 3 |  |
|  | Skewness | | -.438 | .138 |
|  | Kurtosis | | -1.561 | .275 |
| Flixibility | Mean | | 7.29 | .085 |
|  | 95% Confidence Interval for Mean | Lower Bound | 7.12 |  |
|  |  | Upper Bound | 7.46 |  |
|  | 5% Trimmed Mean | | 7.40 |  |
|  | Median | | 7.00 |  |
|  | Variance | | 2.272 |  |
|  | Std. Deviation | | 1.507 |  |
|  | Minimum | | 1 |  |
|  | Maximum | | 9 |  |
|  | Range | | 8 |  |
|  | Interquartile Range | | 3 |  |
|  | Skewness | | -.920 | .138 |
|  | Kurtosis | | .862 | .275 |
| NaturalLight | Mean | | 8.35 | .054 |
|  | 95% Confidence Interval for Mean | Lower Bound | 8.25 |  |
|  |  | Upper Bound | 8.46 |  |
|  | 5% Trimmed Mean | | 8.47 |  |
|  | Median | | 9.00 |  |
|  | Variance | | .904 |  |
|  | Std. Deviation | | .951 |  |
|  | Minimum | | 3 |  |
|  | Maximum | | 9 |  |
|  | Range | | 6 |  |
|  | Interquartile Range | | 1 |  |
|  | Skewness | | -1.796 | .138 |
|  | Kurtosis | | 4.132 | .275 |
| EnteSpace | Mean | | 7.08 | .094 |
|  | 95% Confidence Interval for Mean | Lower Bound | 6.90 |  |
|  |  | Upper Bound | 7.27 |  |
|  | 5% Trimmed Mean | | 7.20 |  |
|  | Median | | 7.00 |  |
|  | Variance | | 2.733 |  |
|  | Std. Deviation | | 1.653 |  |
|  | Minimum | | 1 |  |
|  | Maximum | | 9 |  |
|  | Range | | 8 |  |
|  | Interquartile Range | | 3 |  |
|  | Skewness | | -.843 | .138 |
|  | Kurtosis | | .647 | .275 |
| Storage | Mean | | 6.74 | .099 |
|  | 95% Confidence Interval for Mean | Lower Bound | 6.54 |  |
|  |  | Upper Bound | 6.93 |  |
|  | 5% Trimmed Mean | | 6.85 |  |
|  | Median | | 7.00 |  |
|  | Variance | | 3.069 |  |
|  | Std. Deviation | | 1.752 |  |
|  | Minimum | | 1 |  |
|  | Maximum | | 9 |  |
|  | Range | | 8 |  |
|  | Interquartile Range | | 2 |  |
|  | Skewness | | -.738 | .138 |
|  | Kurtosis | | .387 | .275 |
| Homeoffice | Mean | | 7.30 | .089 |
|  | 95% Confidence Interval for Mean | Lower Bound | 7.13 |  |
|  |  | Upper Bound | 7.48 |  |
|  | 5% Trimmed Mean | | 7.43 |  |
|  | Median | | 8.00 |  |
|  | Variance | | 2.476 |  |
|  | Std. Deviation | | 1.574 |  |
|  | Minimum | | 1 |  |
|  | Maximum | | 9 |  |
|  | Range | | 8 |  |
|  | Interquartile Range | | 2 |  |
|  | Skewness | | -1.175 | .138 |
|  | Kurtosis | | 1.932 | .275 |
| Terrace | Mean | | 7.95 | .079 |
|  | 95% Confidence Interval for Mean | Lower Bound | 7.79 |  |
|  |  | Upper Bound | 8.10 |  |
|  | 5% Trimmed Mean | | 8.10 |  |
|  | Median | | 8.50 |  |
|  | Variance | | 1.968 |  |
|  | Std. Deviation | | 1.403 |  |
|  | Minimum | | 2 |  |
|  | Maximum | | 9 |  |
|  | Range | | 7 |  |
|  | Interquartile Range | | 2 |  |
|  | Skewness | | -1.527 | .138 |
|  | Kurtosis | | 2.267 | .275 |
| Bedroom | Mean | | 7.73 | .090 |
|  | 95% Confidence Interval for Mean | Lower Bound | 7.55 |  |
|  |  | Upper Bound | 7.91 |  |
|  | 5% Trimmed Mean | | 7.91 |  |
|  | Median | | 8.00 |  |
|  | Variance | | 2.525 |  |
|  | Std. Deviation | | 1.589 |  |
|  | Minimum | | 1 |  |
|  | Maximum | | 9 |  |
|  | Range | | 8 |  |
|  | Interquartile Range | | 2 |  |
|  | Skewness | | -1.540 | .138 |
|  | Kurtosis | | 2.487 | .275 |
| Entrance | Mean | | 7.10 | .102 |
|  | 95% Confidence Interval for Mean | Lower Bound | 6.89 |  |
|  |  | Upper Bound | 7.30 |  |
|  | 5% Trimmed Mean | | 7.24 |  |
|  | Median | | 8.00 |  |
|  | Variance | | 3.277 |  |
|  | Std. Deviation | | 1.810 |  |
|  | Minimum | | 1 |  |
|  | Maximum | | 9 |  |
|  | Range | | 8 |  |
|  | Interquartile Range | | 3 |  |
|  | Skewness | | -1.035 | .138 |
|  | Kurtosis | | .858 | .275 |

| **Extreme Values** | | | | |
| --- | --- | --- | --- | --- |
|  | | | Case Number | Value |
| Groups | Highest | 1 | 7 | 4 |
|  |  | 2 | 8 | 4 |
|  |  | 3 | 10 | 4 |
|  |  | 4 | 12 | 4 |
|  |  | 5 | 15 | 4^a^ |
|  | Lowest | 1 | 314 | 1 |
|  |  | 2 | 313 | 1 |
|  |  | 3 | 311 | 1 |
|  |  | 4 | 310 | 1 |
|  |  | 5 | 301 | 1^b^ |
| Flixibility | Highest | 1 | 1 | 9 |
|  |  | 2 | 8 | 9 |
|  |  | 3 | 9 | 9 |
|  |  | 4 | 15 | 9 |
|  |  | 5 | 22 | 9^c^ |
|  | Lowest | 1 | 249 | 1 |
|  |  | 2 | 111 | 2 |
|  |  | 3 | 314 | 3 |
|  |  | 4 | 274 | 3 |
|  |  | 5 | 151 | 3^d^ |
| NaturalLight | Highest | 1 | 1 | 9 |
|  |  | 2 | 7 | 9 |
|  |  | 3 | 9 | 9 |
|  |  | 4 | 10 | 9 |
|  |  | 5 | 12 | 9^c^ |
|  | Lowest | 1 | 11 | 3 |
|  |  | 2 | 190 | 5 |
|  |  | 3 | 176 | 5 |
|  |  | 4 | 149 | 5 |
|  |  | 5 | 90 | 5 |
| EnteSpace | Highest | 1 | 7 | 9 |
|  |  | 2 | 9 | 9 |
|  |  | 3 | 19 | 9 |
|  |  | 4 | 22 | 9 |
|  |  | 5 | 27 | 9^c^ |
|  | Lowest | 1 | 161 | 1 |
|  |  | 2 | 89 | 1 |
|  |  | 3 | 299 | 2 |
|  |  | 4 | 111 | 2 |
|  |  | 5 | 314 | 3^d^ |
| Storage | Highest | 1 | 7 | 9 |
|  |  | 2 | 9 | 9 |
|  |  | 3 | 14 | 9 |
|  |  | 4 | 19 | 9 |
|  |  | 5 | 22 | 9^c^ |
|  | Lowest | 1 | 249 | 1 |
|  |  | 2 | 232 | 1 |
|  |  | 3 | 166 | 1 |
|  |  | 4 | 299 | 2 |
|  |  | 5 | 45 | 2^e^ |
| Homeoffice | Highest | 1 | 7 | 9 |
|  |  | 2 | 9 | 9 |
|  |  | 3 | 10 | 9 |
|  |  | 4 | 12 | 9 |
|  |  | 5 | 14 | 9^c^ |
|  | Lowest | 1 | 293 | 1 |
|  |  | 2 | 249 | 1 |
|  |  | 3 | 232 | 1 |
|  |  | 4 | 299 | 2 |
|  |  | 5 | 45 | 2 |
| Terrace | Highest | 1 | 1 | 9 |
|  |  | 2 | 7 | 9 |
|  |  | 3 | 9 | 9 |
|  |  | 4 | 10 | 9 |
|  |  | 5 | 12 | 9^c^ |
|  | Lowest | 1 | 232 | 2 |
|  |  | 2 | 45 | 2 |
|  |  | 3 | 255 | 3 |
|  |  | 4 | 30 | 3 |
|  |  | 5 | 174 | 4^f^ |
| Bedroom | Highest | 1 | 1 | 9 |
|  |  | 2 | 7 | 9 |
|  |  | 3 | 9 | 9 |
|  |  | 4 | 14 | 9 |
|  |  | 5 | 15 | 9^c^ |
|  | Lowest | 1 | 299 | 1 |
|  |  | 2 | 232 | 1 |
|  |  | 3 | 45 | 2 |
|  |  | 4 | 314 | 3 |
|  |  | 5 | 231 | 3^d^ |
| Entrance | Highest | 1 | 9 | 9 |
|  |  | 2 | 14 | 9 |
|  |  | 3 | 15 | 9 |
|  |  | 4 | 19 | 9 |
|  |  | 5 | 25 | 9^c^ |
|  | Lowest | 1 | 299 | 1 |
|  |  | 2 | 259 | 1 |
|  |  | 3 | 232 | 1 |
|  |  | 4 | 129 | 1 |
|  |  | 5 | 90 | 1 |
| a. Only a partial list of cases with the value 4 are shown in the table of upper extremes. | | | | |
| b. Only a partial list of cases with the value 1 are shown in the table of lower extremes. | | | | |
| c. Only a partial list of cases with the value 9 are shown in the table of upper extremes. | | | | |
| d. Only a partial list of cases with the value 3 are shown in the table of lower extremes. | | | | |
| e. Only a partial list of cases with the value 2 are shown in the table of lower extremes. | | | | |
| f. Only a partial list of cases with the value 4 are shown in the table of lower extremes. | | | | |

**Oneway**

| **Notes** | | |
| --- | --- | --- |
| Output Created | | 10-JUL-2022 20:38:39 |
| Comments | |  |
| Input | Data | C:\Users\Nancy\Documents\Phd\Springer Nature Journal Paper phase 2\Latest-107DATABASE SPSS-Home Features Post COVID 19.sav |
|  | Active Dataset | DataSet3 |
|  | Filter | <none> |
|  | Weight | <none> |
|  | Split File | <none> |
|  | N of Rows in Working Data File | 315 |
| Missing Value Handling | Definition of Missing | User-defined missing values are treated as missing. |
|  | Cases Used | Statistics for each analysis are based on cases with no missing data for any variable in the analysis. |
| Syntax | | ONEWAY Flixibility NaturalLight EnteSpace Storage Homeoffice Terrace Bedroom Entrance BY Groups /ES=OVERALL /STATISTICS DESCRIPTIVES /MISSING ANALYSIS /CRITERIA=CILEVEL(0.95) /POSTHOC=TUKEY ALPHA(0.05). |
| Resources | Processor Time | 00:00:00.03 |
|  | Elapsed Time | 00:00:00.13 |

| **Descriptives** | | | | | | | | |
| --- | --- | --- | --- | --- | --- | --- | --- | --- |
|  | | N | Mean | Std. Deviation | Std. Error | 95% Confidence Interval for Mean | | Minimum |
|  |  |  |  |  |  | Lower Bound | Upper Bound |  |
| Flixibility | Design | 87 | 7.26 | 1.490 | .160 | 6.95 | 7.58 | 3 |
|  | Construction | 35 | 6.94 | 1.878 | .317 | 6.30 | 7.59 | 1 |
|  | Real estate | 39 | 7.36 | 1.423 | .228 | 6.90 | 7.82 | 4 |
|  | Users | 152 | 7.38 | 1.446 | .117 | 7.15 | 7.61 | 3 |
|  | Total | 313 | 7.30 | 1.508 | .085 | 7.13 | 7.46 | 1 |
| NaturalLight | Design | 86 | 8.38 | .910 | .098 | 8.19 | 8.58 | 5 |
|  | Construction | 35 | 8.43 | .917 | .155 | 8.11 | 8.74 | 6 |
|  | Real estate | 39 | 8.15 | 1.113 | .178 | 7.79 | 8.51 | 5 |
|  | Users | 153 | 8.41 | .831 | .067 | 8.28 | 8.54 | 5 |
|  | Total | 313 | 8.37 | .901 | .051 | 8.27 | 8.47 | 5 |
| EnteSpace | Design | 87 | 7.16 | 1.663 | .178 | 6.81 | 7.52 | 1 |
|  | Construction | 36 | 6.75 | 2.048 | .341 | 6.06 | 7.44 | 2 |
|  | Real estate | 39 | 6.41 | 1.831 | .293 | 5.82 | 7.00 | 1 |
|  | Users | 153 | 7.29 | 1.446 | .117 | 7.06 | 7.53 | 2 |
|  | Total | 315 | 7.09 | 1.654 | .093 | 6.90 | 7.27 | 1 |
| Storage | Design | 87 | 6.77 | 1.515 | .162 | 6.45 | 7.09 | 3 |
|  | Construction | 36 | 6.36 | 2.072 | .345 | 5.66 | 7.06 | 1 |
|  | Real estate | 39 | 6.38 | 1.815 | .291 | 5.80 | 6.97 | 1 |
|  | Users | 153 | 6.90 | 1.769 | .143 | 6.62 | 7.18 | 1 |
|  | Total | 315 | 6.74 | 1.751 | .099 | 6.55 | 6.93 | 1 |
| Homeoffice | Design | 87 | 7.30 | 1.415 | .152 | 7.00 | 7.60 | 3 |
|  | Construction | 36 | 6.69 | 1.818 | .303 | 6.08 | 7.31 | 1 |
|  | Real estate | 39 | 7.05 | 1.621 | .260 | 6.53 | 7.58 | 3 |
|  | Users | 153 | 7.54 | 1.547 | .125 | 7.30 | 7.79 | 1 |
|  | Total | 315 | 7.32 | 1.572 | .089 | 7.14 | 7.49 | 1 |
| Terrace | Design | 87 | 7.85 | 1.394 | .149 | 7.55 | 8.15 | 3 |
|  | Construction | 36 | 7.69 | 1.636 | .273 | 7.14 | 8.25 | 3 |
|  | Real estate | 39 | 7.87 | 1.454 | .233 | 7.40 | 8.34 | 4 |
|  | Users | 153 | 8.10 | 1.327 | .107 | 7.89 | 8.31 | 2 |
|  | Total | 315 | 7.96 | 1.400 | .079 | 7.80 | 8.11 | 2 |
| Bedroom | Design | 87 | 7.84 | 1.421 | .152 | 7.54 | 8.14 | 3 |
|  | Construction | 36 | 7.42 | 1.645 | .274 | 6.86 | 7.97 | 3 |
|  | Real estate | 39 | 7.62 | 1.632 | .261 | 7.09 | 8.14 | 3 |
|  | Users | 153 | 7.80 | 1.652 | .134 | 7.53 | 8.06 | 1 |
|  | Total | 315 | 7.74 | 1.586 | .089 | 7.57 | 7.92 | 1 |
| Entrance | Design | 87 | 7.60 | 1.660 | .178 | 7.24 | 7.95 | 1 |
|  | Construction | 36 | 6.94 | 1.706 | .284 | 6.37 | 7.52 | 3 |
|  | Real estate | 39 | 6.79 | 1.908 | .306 | 6.18 | 7.41 | 2 |
|  | Users | 153 | 6.93 | 1.856 | .150 | 6.64 | 7.23 | 1 |
|  | Total | 315 | 7.10 | 1.812 | .102 | 6.90 | 7.30 | 1 |

| **Descriptives** | | |
| --- | --- | --- |
|  | | Maximum |
|  |  |  |
| Flixibility | Design | 9 |
|  | Construction | 9 |
|  | Real estate | 9 |
|  | Users | 9 |
|  | Total | 9 |
| NaturalLight | Design | 9 |
|  | Construction | 9 |
|  | Real estate | 9 |
|  | Users | 9 |
|  | Total | 9 |
| EnteSpace | Design | 9 |
|  | Construction | 9 |
|  | Real estate | 9 |
|  | Users | 9 |
|  | Total | 9 |
| Storage | Design | 9 |
|  | Construction | 9 |
|  | Real estate | 9 |
|  | Users | 9 |
|  | Total | 9 |
| Homeoffice | Design | 9 |
|  | Construction | 9 |
|  | Real estate | 9 |
|  | Users | 9 |
|  | Total | 9 |
| Terrace | Design | 9 |
|  | Construction | 9 |
|  | Real estate | 9 |
|  | Users | 9 |
|  | Total | 9 |
| Bedroom | Design | 9 |
|  | Construction | 9 |
|  | Real estate | 9 |
|  | Users | 9 |
|  | Total | 9 |
| Entrance | Design | 9 |
|  | Construction | 9 |
|  | Real estate | 9 |
|  | Users | 9 |
|  | Total | 9 |

| **ANOVA** | | | | | | |
| --- | --- | --- | --- | --- | --- | --- |
|  | | Sum of Squares | df | Mean Square | F | Sig. |
| Flixibility | Between Groups | 5.719 | 3 | 1.906 | .837 | .474 |
|  | Within Groups | 703.648 | 309 | 2.277 |  |  |
|  | Total | 709.367 | 312 |  |  |  |
| NaturalLight | Between Groups | 2.221 | 3 | .740 | .911 | .436 |
|  | Within Groups | 251.044 | 309 | .812 |  |  |
|  | Total | 253.265 | 312 |  |  |  |
| EnteSpace | Between Groups | 28.988 | 3 | 9.663 | 3.622 | .013 |
|  | Within Groups | 829.698 | 311 | 2.668 |  |  |
|  | Total | 858.686 | 314 |  |  |  |
| Storage | Between Groups | 14.186 | 3 | 4.729 | 1.551 | .201 |
|  | Within Groups | 948.468 | 311 | 3.050 |  |  |
|  | Total | 962.654 | 314 |  |  |  |
| Homeoffice | Between Groups | 24.514 | 3 | 8.171 | 3.381 | .019 |
|  | Within Groups | 751.740 | 311 | 2.417 |  |  |
|  | Total | 776.254 | 314 |  |  |  |
| Terrace | Between Groups | 6.793 | 3 | 2.264 | 1.157 | .326 |
|  | Within Groups | 608.585 | 311 | 1.957 |  |  |
|  | Total | 615.378 | 314 |  |  |  |
| Bedroom | Between Groups | 5.725 | 3 | 1.908 | .757 | .519 |
|  | Within Groups | 784.447 | 311 | 2.522 |  |  |
|  | Total | 790.171 | 314 |  |  |  |
| Entrance | Between Groups | 30.235 | 3 | 10.078 | 3.133 | .026 |
|  | Within Groups | 1000.514 | 311 | 3.217 |  |  |
|  | Total | 1030.749 | 314 |  |  |  |

| **ANOVA Effect Sizes**^a,b^ | | | | |
| --- | --- | --- | --- | --- |
|  | | Point Estimate | 95% Confidence Interval | |
|  |  |  | Lower | Upper |
| Flixibility | Eta-squared | .008 | .000 | .030 |
|  | Epsilon-squared | -.002 | -.010 | .020 |
|  | Omega-squared Fixed-effect | -.002 | -.010 | .020 |
|  | Omega-squared Random-effect | -.001 | -.003 | .007 |
| NaturalLight | Eta-squared | .009 | .000 | .031 |
|  | Epsilon-squared | -.001 | -.010 | .022 |
|  | Omega-squared Fixed-effect | -.001 | -.010 | .022 |
|  | Omega-squared Random-effect | .000 | -.003 | .007 |
| EnteSpace | Eta-squared | .034 | .001 | .074 |
|  | Epsilon-squared | .024 | -.008 | .065 |
|  | Omega-squared Fixed-effect | .024 | -.008 | .065 |
|  | Omega-squared Random-effect | .008 | -.003 | .023 |
| Storage | Eta-squared | .015 | .000 | .043 |
|  | Epsilon-squared | .005 | -.010 | .034 |
|  | Omega-squared Fixed-effect | .005 | -.010 | .034 |
|  | Omega-squared Random-effect | .002 | -.003 | .012 |
| Homeoffice | Eta-squared | .032 | .001 | .071 |
|  | Epsilon-squared | .022 | -.009 | .062 |
|  | Omega-squared Fixed-effect | .022 | -.009 | .062 |
|  | Omega-squared Random-effect | .008 | -.003 | .021 |
| Terrace | Eta-squared | .011 | .000 | .036 |
|  | Epsilon-squared | .001 | -.010 | .027 |
|  | Omega-squared Fixed-effect | .001 | -.010 | .027 |
|  | Omega-squared Random-effect | .000 | -.003 | .009 |
| Bedroom | Eta-squared | .007 | .000 | .028 |
|  | Epsilon-squared | -.002 | -.010 | .018 |
|  | Omega-squared Fixed-effect | -.002 | -.010 | .018 |
|  | Omega-squared Random-effect | -.001 | -.003 | .006 |
| Entrance | Eta-squared | .029 | .000 | .068 |
|  | Epsilon-squared | .020 | -.010 | .059 |
|  | Omega-squared Fixed-effect | .020 | -.010 | .058 |
|  | Omega-squared Random-effect | .007 | -.003 | .020 |
| a. Eta-squared and Epsilon-squared are estimated based on the fixed-effect model. | | | | |
| b. Negative but less biased estimates are retained, not rounded to zero. | | | | |

**Post Hoc Tests**

| **Multiple Comparisons** | | | | | | | |
| --- | --- | --- | --- | --- | --- | --- | --- |
| Tukey HSD | | | | | | | |
| Dependent Variable | (I) Groups | (J) Groups | Mean Difference (I-J) | Std. Error | Sig. | 95% Confidence Interval | |
|  |  |  |  |  |  | Lower Bound | Upper Bound |
| Flixibility | Design | Construction | .322 | .302 | .711 | -.46 | 1.10 |
|  |  | Real estate | -.095 | .291 | .988 | -.85 | .66 |
|  |  | Users | -.117 | .203 | .939 | -.64 | .41 |
|  | Construction | Design | -.322 | .302 | .711 | -1.10 | .46 |
|  |  | Real estate | -.416 | .351 | .637 | -1.32 | .49 |
|  |  | Users | -.439 | .283 | .409 | -1.17 | .29 |
|  | Real estate | Design | .095 | .291 | .988 | -.66 | .85 |
|  |  | Construction | .416 | .351 | .637 | -.49 | 1.32 |
|  |  | Users | -.023 | .271 | 1.000 | -.72 | .68 |
|  | Users | Design | .117 | .203 | .939 | -.41 | .64 |
|  |  | Construction | .439 | .283 | .409 | -.29 | 1.17 |
|  |  | Real estate | .023 | .271 | 1.000 | -.68 | .72 |
| NaturalLight | Design | Construction | -.045 | .181 | .995 | -.51 | .42 |
|  |  | Real estate | .230 | .174 | .550 | -.22 | .68 |
|  |  | Users | -.028 | .121 | .996 | -.34 | .29 |
|  | Construction | Design | .045 | .181 | .995 | -.42 | .51 |
|  |  | Real estate | .275 | .210 | .558 | -.27 | .82 |
|  |  | Users | .017 | .169 | 1.000 | -.42 | .45 |
|  | Real estate | Design | -.230 | .174 | .550 | -.68 | .22 |
|  |  | Construction | -.275 | .210 | .558 | -.82 | .27 |
|  |  | Users | -.258 | .162 | .383 | -.68 | .16 |
|  | Users | Design | .028 | .121 | .996 | -.29 | .34 |
|  |  | Construction | -.017 | .169 | 1.000 | -.45 | .42 |
|  |  | Real estate | .258 | .162 | .383 | -.16 | .68 |
| EnteSpace | Design | Construction | .411 | .324 | .583 | -.43 | 1.25 |
|  |  | Real estate | .751 | .315 | .082 | -.06 | 1.56 |
|  |  | Users | -.133 | .219 | .930 | -.70 | .43 |
|  | Construction | Design | -.411 | .324 | .583 | -1.25 | .43 |
|  |  | Real estate | .340 | .378 | .805 | -.64 | 1.31 |
|  |  | Users | -.544 | .303 | .276 | -1.33 | .24 |
|  | Real estate | Design | -.751 | .315 | .082 | -1.56 | .06 |
|  |  | Construction | -.340 | .378 | .805 | -1.31 | .64 |
|  |  | Users | -.884^*^ | .293 | .015 | -1.64 | -.13 |
|  | Users | Design | .133 | .219 | .930 | -.43 | .70 |
|  |  | Construction | .544 | .303 | .276 | -.24 | 1.33 |
|  |  | Real estate | .884^*^ | .293 | .015 | .13 | 1.64 |
| Storage | Design | Construction | .409 | .346 | .639 | -.48 | 1.30 |
|  |  | Real estate | .385 | .337 | .662 | -.48 | 1.25 |
|  |  | Users | -.132 | .234 | .943 | -.74 | .47 |
|  | Construction | Design | -.409 | .346 | .639 | -1.30 | .48 |
|  |  | Real estate | -.024 | .404 | 1.000 | -1.07 | 1.02 |
|  |  | Users | -.541 | .323 | .340 | -1.38 | .29 |
|  | Real estate | Design | -.385 | .337 | .662 | -1.25 | .48 |
|  |  | Construction | .024 | .404 | 1.000 | -1.02 | 1.07 |
|  |  | Users | -.517 | .313 | .351 | -1.33 | .29 |
|  | Users | Design | .132 | .234 | .943 | -.47 | .74 |
|  |  | Construction | .541 | .323 | .340 | -.29 | 1.38 |
|  |  | Real estate | .517 | .313 | .351 | -.29 | 1.33 |
| Homeoffice | Design | Construction | .604 | .308 | .205 | -.19 | 1.40 |
|  |  | Real estate | .248 | .300 | .842 | -.53 | 1.02 |
|  |  | Users | -.244 | .209 | .648 | -.78 | .30 |
|  | Construction | Design | -.604 | .308 | .205 | -1.40 | .19 |
|  |  | Real estate | -.357 | .359 | .754 | -1.28 | .57 |
|  |  | Users | -.848^*^ | .288 | .018 | -1.59 | -.10 |
|  | Real estate | Design | -.248 | .300 | .842 | -1.02 | .53 |
|  |  | Construction | .357 | .359 | .754 | -.57 | 1.28 |
|  |  | Users | -.491 | .279 | .294 | -1.21 | .23 |
|  | Users | Design | .244 | .209 | .648 | -.30 | .78 |
|  |  | Construction | .848^*^ | .288 | .018 | .10 | 1.59 |
|  |  | Real estate | .491 | .279 | .294 | -.23 | 1.21 |
| Terrace | Design | Construction | .156 | .277 | .943 | -.56 | .87 |
|  |  | Real estate | -.021 | .270 | 1.000 | -.72 | .68 |
|  |  | Users | -.247 | .188 | .552 | -.73 | .24 |
|  | Construction | Design | -.156 | .277 | .943 | -.87 | .56 |
|  |  | Real estate | -.177 | .323 | .947 | -1.01 | .66 |
|  |  | Users | -.404 | .259 | .405 | -1.07 | .27 |
|  | Real estate | Design | .021 | .270 | 1.000 | -.68 | .72 |
|  |  | Construction | .177 | .323 | .947 | -.66 | 1.01 |
|  |  | Users | -.226 | .251 | .804 | -.87 | .42 |
|  | Users | Design | .247 | .188 | .552 | -.24 | .73 |
|  |  | Construction | .404 | .259 | .405 | -.27 | 1.07 |
|  |  | Real estate | .226 | .251 | .804 | -.42 | .87 |
| Bedroom | Design | Construction | .422 | .315 | .537 | -.39 | 1.24 |
|  |  | Real estate | .224 | .306 | .885 | -.57 | 1.01 |
|  |  | Users | .042 | .213 | .997 | -.51 | .59 |
|  | Construction | Design | -.422 | .315 | .537 | -1.24 | .39 |
|  |  | Real estate | -.199 | .367 | .949 | -1.15 | .75 |
|  |  | Users | -.381 | .294 | .567 | -1.14 | .38 |
|  | Real estate | Design | -.224 | .306 | .885 | -1.01 | .57 |
|  |  | Construction | .199 | .367 | .949 | -.75 | 1.15 |
|  |  | Users | -.182 | .285 | .919 | -.92 | .55 |
|  | Users | Design | -.042 | .213 | .997 | -.59 | .51 |
|  |  | Construction | .381 | .294 | .567 | -.38 | 1.14 |
|  |  | Real estate | .182 | .285 | .919 | -.55 | .92 |
| Entrance | Design | Construction | .653 | .355 | .258 | -.26 | 1.57 |
|  |  | Real estate | .803 | .346 | .095 | -.09 | 1.70 |
|  |  | Users | .663^*^ | .241 | .032 | .04 | 1.29 |
|  | Construction | Design | -.653 | .355 | .258 | -1.57 | .26 |
|  |  | Real estate | .150 | .415 | .984 | -.92 | 1.22 |
|  |  | Users | .010 | .332 | 1.000 | -.85 | .87 |
|  | Real estate | Design | -.803 | .346 | .095 | -1.70 | .09 |
|  |  | Construction | -.150 | .415 | .984 | -1.22 | .92 |
|  |  | Users | -.140 | .322 | .973 | -.97 | .69 |
|  | Users | Design | -.663^*^ | .241 | .032 | -1.29 | -.04 |
|  |  | Construction | -.010 | .332 | 1.000 | -.87 | .85 |
|  |  | Real estate | .140 | .322 | .973 | -.69 | .97 |
| *. The mean difference is significant at the 0.05 level. | | | | | | | |

**Homogeneous Subsets**

| **Flixibility** | | |
| --- | --- | --- |
| Tukey HSD^a,b^ | | |
| Groups | N | Subset for alpha = 0.05 |
|  |  | 1 |
| Construction | 35 | 6.94 |
| Design | 87 | 7.26 |
| Real estate | 39 | 7.36 |
| Users | 152 | 7.38 |
| Sig. |  | .421 |
| Means for groups in homogeneous subsets are displayed. | | |
| a. Uses Harmonic Mean Sample Size = 55.336. | | |
| b. The group sizes are unequal. The harmonic mean of the group sizes is used. Type I error levels are not guaranteed. | | |

| **NaturalLight** | | |
| --- | --- | --- |
| Tukey HSD^a,b^ | | |
| Groups | N | Subset for alpha = 0.05 |
|  |  | 1 |
| Real estate | 39 | 8.15 |
| Design | 86 | 8.38 |
| Users | 153 | 8.41 |
| Construction | 35 | 8.43 |
| Sig. |  | .379 |
| Means for groups in homogeneous subsets are displayed. | | |
| a. Uses Harmonic Mean Sample Size = 55.267. | | |
| b. The group sizes are unequal. The harmonic mean of the group sizes is used. Type I error levels are not guaranteed. | | |

| **EnteSpace** | | | |
| --- | --- | --- | --- |
| Tukey HSD^a,b^ | | | |
| Groups | N | Subset for alpha = 0.05 | |
|  |  | 1 | 2 |
| Real estate | 39 | 6.41 |  |
| Construction | 36 | 6.75 | 6.75 |
| Design | 87 | 7.16 | 7.16 |
| Users | 153 |  | 7.29 |
| Sig. |  | .073 | .294 |
| Means for groups in homogeneous subsets are displayed. | | | |
| a. Uses Harmonic Mean Sample Size = 55.984. | | | |
| b. The group sizes are unequal. The harmonic mean of the group sizes is used. Type I error levels are not guaranteed. | | | |

| **Storage** | | |
| --- | --- | --- |
| Tukey HSD^a,b^ | | |
| Groups | N | Subset for alpha = 0.05 |
|  |  | 1 |
| Construction | 36 | 6.36 |
| Real estate | 39 | 6.38 |
| Design | 87 | 6.77 |
| Users | 153 | 6.90 |
| Sig. |  | .359 |
| Means for groups in homogeneous subsets are displayed. | | |
| a. Uses Harmonic Mean Sample Size = 55.984. | | |
| b. The group sizes are unequal. The harmonic mean of the group sizes is used. Type I error levels are not guaranteed. | | |

| **Homeoffice** | | | |
| --- | --- | --- | --- |
| Tukey HSD^a,b^ | | | |
| Groups | N | Subset for alpha = 0.05 | |
|  |  | 1 | 2 |
| Construction | 36 | 6.69 |  |
| Real estate | 39 | 7.05 | 7.05 |
| Design | 87 | 7.30 | 7.30 |
| Users | 153 |  | 7.54 |
| Sig. |  | .170 | .341 |
| Means for groups in homogeneous subsets are displayed. | | | |
| a. Uses Harmonic Mean Sample Size = 55.984. | | | |
| b. The group sizes are unequal. The harmonic mean of the group sizes is used. Type I error levels are not guaranteed. | | | |

| **Terrace** | | |
| --- | --- | --- |
| Tukey HSD^a,b^ | | |
| Groups | N | Subset for alpha = 0.05 |
|  |  | 1 |
| Construction | 36 | 7.69 |
| Design | 87 | 7.85 |
| Real estate | 39 | 7.87 |
| Users | 153 | 8.10 |
| Sig. |  | .423 |
| Means for groups in homogeneous subsets are displayed. | | |
| a. Uses Harmonic Mean Sample Size = 55.984. | | |
| b. The group sizes are unequal. The harmonic mean of the group sizes is used. Type I error levels are not guaranteed. | | |

| **Bedroom** | | |
| --- | --- | --- |
| Tukey HSD^a,b^ | | |
| Groups | N | Subset for alpha = 0.05 |
|  |  | 1 |
| Construction | 36 | 7.42 |
| Real estate | 39 | 7.62 |
| Users | 153 | 7.80 |
| Design | 87 | 7.84 |
| Sig. |  | .496 |
| Means for groups in homogeneous subsets are displayed. | | |
| a. Uses Harmonic Mean Sample Size = 55.984. | | |
| b. The group sizes are unequal. The harmonic mean of the group sizes is used. Type I error levels are not guaranteed. | | |

| **Entrance** | | |
| --- | --- | --- |
| Tukey HSD^a,b^ | | |
| Groups | N | Subset for alpha = 0.05 |
|  |  | 1 |
| Real estate | 39 | 6.79 |
| Users | 153 | 6.93 |
| Construction | 36 | 6.94 |
| Design | 87 | 7.60 |
| Sig. |  | .085 |
| Means for groups in homogeneous subsets are displayed. | | |
| a. Uses Harmonic Mean Sample Size = 55.984. | | |
| b. The group sizes are unequal. The harmonic mean of the group sizes is used. Type I error levels are not guaranteed. | | |
