# Supplementary material for: Home design features post-COVID-19
Source: J. Eng. Appl. Sci. 2022 Sep 30;69(1):87. doi: 10.1186/s44147-022-00142-z (PMC9523634; doi:10.1186/s44147-022-00142-z)
Supplement: Supplementary file 3 — Additional file 3. [file 44147_2022_142_MOESM3_ESM.docx]

| id | IP | Loc | Class | Occ | Group | Flexibility | Natural Light | EntertSpace | Storage | HomeOffice | Terrace | Bedroom | Entrance |
| --- | --- | --- | --- | --- | --- | --- | --- | --- | --- | --- | --- | --- | --- |
| 1 | 102.184.248.122 | تجمع خامس | 1 | Architect | 1 | 9 | 9 | 6 | 6 | 7 | 9 | 9 | 8 |
| 2 | 102.184.252.128 | الشروق | 1 | Architect | 1 | 8 | 8 | 5 | 5 | 6 | 8 | 8 | 8 |
| 3 | 102.184.47.2 | مصر الجديد� | 2 | Contractor | 2 | 5 | 8 | 3 | 3 | 6 | 8 | 7 | 6 |
| 4 | 102.186.205.41 | ٦ اكتوبر | 2 | Realtor | 3 | 6 | 6 | 7 | 6 | 6 | 4 | 6 | 5 |
| 5 | 102.186.208.98 | مدينة نصر | 2 | Urban Planner | 1 | 7 | 8 | 7 | 6 | 7 | 8 | 7 | 7 |
| 6 | 102.190.134.56 | مدينة الشر� | 3 | Contractor | 2 | 8 | 8 | 5 | 5 | 6 | 7 | 8 | 8 |
| 7 | 102.40.123.191 | مدينة العب� | 3 | Resident | 4 | 8 | 9 | 9 | 9 | 9 | 9 | 9 | 8 |
| 8 | 102.42.149.165 | العبور | 2 | resident | 4 | 9 | 8 | 4 | 5 | 6 | 7 | 8 | 6 |
| 9 | 102.43.120.228 | المعادي | 2 | Architect | 1 | 9 | 9 | 9 | 9 | 9 | 9 | 9 | 9 |
| 10 | 102.43.75.34 | ٦ اكتوبر | 1 | resident | 4 | 8 | 9 | 8 | 4 | 9 | 9 | 8 | 4 |
| 11 | 102.43.80.176 | شبرا مصر | 2 | Contractor | 2 | 5 |  | 5 | 5 | 5 | 5 | 5 | 5 |
| 12 | 102.44.226.113 | ٦ أكتوبر | 1 | resident | 4 | 6 | 9 | 7 | 4 | 9 | 9 | 8 | 8 |
| 13 | 102.44.4.212 | حدائق القب� | 3 | Architect | 1 | 7 | 9 | 7 | 6 | 7 | 6 | 7 | 5 |
| 14 | 102.46.84.58 | N/A | 2 | Architect | 1 | 5 | 9 | 5 | 9 | 9 | 9 | 9 | 9 |
| 15 | 102.56.178.20 | التجمع الخ� | 2 | resident | 4 | 9 | 9 | 8 | 8 | 8 | 9 | 9 | 9 |
| 16 | 102.57.126.200 | فيصل | 3 | resident | 4 | 6 | 8 | 7 | 6 | 7 | 8 | 7 | 5 |
| 17 | 102.57.142.42 | الرحاب | 1 | resident | 4 | 7 | 9 | 8 | 8 | 8 | 8 | 8 | 7 |
| 18 | 102.57.191.186 | N/A | 3 | resident | 4 | 7 | 9 | 7 | 7 | 9 | 9 | 9 | 8 |
| 19 | 102.57.234.209 | المعادي | 3 | resident | 4 | 8 | 9 | 9 | 9 | 9 | 9 | 9 | 9 |
| 20 | 102.57.58.178 | مدينة المس� | 2 | resident | 4 | 6 | 8 | 6 | 7 | 5 | 6 | 9 | 5 |
| 21 | 102.58.13.138 | التجمع الخ� | 1 | resident | 4 | 6 | 9 | 8 | 6 | 9 | 9 | 8 | 7 |
| 22 | 102.58.192.118 | محافظة الج� | 1 | resident | 4 | 9 | 9 | 9 | 9 | 9 | 9 | 9 | 7 |
| 23 | 102.58.60.98 | التجمع الخ� | 1 | resident | 4 | 7 | 7 | 5 | 6 | 5 | 9 | 8 | 5 |
| 24 | 102.59.124.196 | مصر الجديد� | 1 | resident | 4 | 8 | 7 | 8 | 6 | 8 | 9 | 8 | 7 |
| 25 | 102.59.150.15 | المعادى | 2 | Developer | 3 | 7 | 9 | 8 | 7 | 8 | 9 | 9 | 9 |
| 26 | 102.59.155.242 | الرحاب | 1 | resident | 4 | 8 | 8 | 6 | 3 | 7 | 8 | 8 | 6 |
| 27 | 102.59.214.68 | التجمع | 3 | resident | 4 | 9 | 9 | 9 | 9 | 9 | 9 | 9 | 9 |
| 28 | 102.59.77.201 | N/A | 2 | resident | 4 | 6 | 8 | 7 | 7 | 6 | 9 | 7 | 6 |
| 29 | 102.60.108.157 | النرجس عما� | 2 | Resident | 4 | 9 | 9 | 9 | 9 | 9 | 9 | 9 | 6 |
| 30 | 102.60.197.15 | الرحاب | 2 | Civil Engineer | 2 | 8 | 8 | 3 | 2 | 4 | 3 | 3 | 3 |
| 31 | 102.60.240.138 | القاهره ال� | 1 | resident | 4 | 9 | 7 | 8 | 6 | 8 | 8 | 5 | 6 |
| 32 | 102.61.150.196 | الهرم | 3 | Architect | 1 | 8 | 8 | 6 | 5 | 5 | 5 | 5 | 7 |
| 33 | 102.61.183.11 | ٦ اكتوبر | 2 | resident | 4 | 5 | 9 | 7 | 5 | 7 | 7 | 9 | 7 |
| 34 | 102.61.243.218 | التجمع الا� | 2 | resident | 4 | 7 | 9 | 7 | 7 | 7 | 9 | 7 | 7 |
| 35 | 102.61.46.253 | التجمع الخ� | 2 | Developer | 3 | 9 | 9 | 7 | 7 | 9 | 9 | 9 | 9 |
| 36 | 102.61.77.61 | المقطم | 2 | resident | 4 | 9 | 9 | 9 | 9 | 9 | 9 | 9 | 9 |
| 37 | 102.61.92.213 | N/A | 2 | resident | 4 | 7 | 8 | 6 | 7 | 7 | 6 | 8 | 5 |
| 38 | 102.62.199.50 | التجمع الخ� | 1 | resident | 4 | 9 | 8 | 5 | 5 | 5 | 8 | 5 | 5 |
| 39 | 102.62.33.65 | القاهرة ال� | 2 | Developer | 3 | 6 | 7 | 7 | 5 | 8 | 9 | 7 | 5 |
| 40 | 102.62.4.220 | التجمع الخ� | 1 | resident | 4 | 8 | 9 | 8 | 6 | 8 | 8 | 9 | 6 |
| 41 | 102.62.44.46 | التجمع الخ� | 1 | resident | 4 | 7 | 8 | 7 | 7 | 8 | 8 | 9 | 7 |
| 42 | 102.62.89.248 | مدينة نصر | 2 | Interior Designer | 1 | 8 | 9 | 6 | 7 | 9 | 9 | 9 | 9 |
| 43 | 102.63.161.70 | المعادي | 2 | Civil Engineer | 2 | 9 | 9 | 9 | 7 | 5 | 9 | 9 | 9 |
| 44 | 102.63.188.220 | N/A | 3 | Resident | 4 | 5 | 9 | 7 | 7 | 8 | 7 | 6 | 7 |
| 45 | 102.63.33.77 | القاهرة ال� | 1 | resident | 4 | 7 | 7 | 7 | 2 | 2 | 2 | 2 | 2 |
| 46 | 102.63.62.122 | التجمع الخ� | 2 | resident | 4 | 7 | 8 | 6 | 6 | 8 | 7 | 8 | 8 |
| 47 | 102.63.97.89 | البنفسج ٩ | 2 | resident | 4 | 8 | 8 | 9 | 9 | 9 | 8 | 9 | 8 |
| 48 | 105.180.178.39 | المهندسين | 2 | resident | 4 | 8 | 7 | 7 | 7 | 7 | 8 | 6 | 4 |
| 49 | 105.183.215.119 | مصر الجديد� | 2 | Contractor | 2 | 7 | 9 | 9 | 7 | 7 | 9 | 7 | 9 |
| 50 | 105.183.85.6 | مدينة نصر | 2 | Contractor | 2 | 9 | 9 | 5 | 5 | 5 | 8 | 6 | 6 |
| 51 | 105.193.58.124 | شبرا مصر | 2 | Architect | 1 | 7 | 9 | 7 | 7 | 8 | 9 | 9 | 9 |
| 52 | 105.193.80.200 | مدينه نصر | 2 | resident | 4 | 8 | 9 | 6 | 5 | 5 | 5 | 8 | 8 |
| 53 | 105.193.93.16 | التجمع الخ� | 1 | resident | 4 | 6 | 8 | 6 | 5 | 6 | 7 | 6 | 5 |
| 54 | 105.195.208.229 | أكتوبر | 3 | Contractor | 2 | 8 | 9 | 8 | 8 | 9 | 9 | 9 | 8 |
| 55 | 105.195.4.63 | مصر الجديد� | 4 | Architect | 1 | 7 | 9 | 9 | 8 | 7 | 9 | 9 | 8 |
| 56 | 105.196.67.35 | التجمع الخ� | 2 | resident | 4 | 7 | 7 | 6 | 6 | 7 | 8 | 9 | 7 |
| 57 | 105.200.153.78 | مدينة نصر | 2 | resident | 4 | 6 | 9 | 8 | 8 | 8 | 9 | 9 | 4 |
| 58 | 105.201.8.140 | مدينة نصر | 2 | resident | 4 | 5 | 8 | 8 | 8 | 8 | 8 | 7 | 5 |
| 59 | 105.204.216.124 | الرحاب | 1 | resident | 4 | 8 | 8 | 8 | 8 | 8 | 7 | 8 | 8 |
| 60 | 105.204.216.197 | مدينتي | 2 | resident | 4 | 5 | 8 | 7 | 7 | 7 | 9 | 9 | 7 |
| 61 | 105.204.231.114 | الشيخ زايد | 1 | resident | 4 | 9 | 9 | 9 | 5 | 7 | 9 | 9 | 4 |
| 62 | 105.235.136.173 | | 2 | Civil Engineer | 2 | 7 | 9 | 8 | 7 | 8 | 8 | 6 | 6 |
| 63 | 105.39.183.57 | القاهره ال� | 2 | resident | 4 | 9 | 9 | 7 | 6 | 7 | 9 | 9 | 8 |
| 64 | 105.46.203.216 | مصر الجديد� | 2 | resident | 4 | 9 | 9 | 9 | 9 | 9 | 9 | 9 | 9 |
| 65 | 105.46.233.151 | مدينة الشر� | 2 | resident | 4 | 9 | 9 | 9 | 9 | 9 | 9 | 9 | 9 |
| 66 | 105.46.50.5 | الشروق | 1 | Architect | 1 | 7 | 8 | 7 | 6 | 7 | 7 | 8 | 8 |
| 67 | 105.92.10.46 | القناطر ال� | 2 | resident | 4 | 4 | 7 | 7 | 7 | 9 | 8 | 5 | 5 |
| 68 | 105.92.11.92 | أكتوبر الج | 3 | resident | 4 | 6 | 8 | 7 | 7 | 7 | 8 | 8 | 6 |
| 69 | 141.164.212.89 | N/A | 2 | Civil Engineer | 2 | 6 | 6 | 4 | 4 | 5 | 6 | 6 | 5 |
| 70 | 154.129.24.178 | الشيخ زايد | 2 | Architect | 1 | 9 | 9 | 8 | 7 | 9 | 9 | 9 | 9 |
| 71 | 154.132.199.249 | التجمع الخ� | 2 | resident | 4 | 8 | 9 | 9 | 7 | 9 | 9 | 9 | 9 |
| 72 | 154.135.142.172 | 6 أكتوبر | 1 | Civil Engineer | 2 | 9 | 9 | 9 | 9 | 9 | 9 | 9 | 9 |
| 73 | 154.135.6.120 | Heliopolis | 2 | Civil Engineer | 2 | 5 | 9 | 6 | 6 | 6 | 6 | 6 | 5 |
| 74 | 154.143.238.212 | القاهرة ال� | 2 | Contractor | 2 | 5 | 9 | 7 | 7 | 7 | 9 | 9 | 9 |
| 75 | 154.143.39.241 | الدقي | 2 | Realtor | 3 | 9 | 7 | 5 | 5 | 8 | 8 | 8 | 8 |
| 76 | 154.176.252.99 | الهرم | 3 | Architect | 1 | 9 | 9 | 9 | 9 | 9 | 9 | 9 | 9 |
| 77 | 154.183.105.128 | مدينه نصر | 3 | Realtor | 3 | 5 | 8 | 7 | 7 | 8 | 6 | 6 | 7 |
| 78 | 154.185.186.116 | N/A | 2 | Architect | 1 | 5 | 7 | 7 | 7 | 6 | 9 | 9 | 9 |
| 79 | 154.187.67.230 | الشروق | 3 | Architect | 1 | 7 | 6 | 6 | 4 | 5 | 9 | 9 | 9 |
| 80 | 154.190.207.19 | مدينة الشي� | 1 | resident | 4 | 8 | 9 | 9 | 7 | 9 | 9 | 9 | 9 |
| 81 | 154.190.218.89 | الهرم | 3 | resident | 4 | 6 | 8 | 7 | 5 | 8 | 7 | 6 | 6 |
| 82 | 154.190.253.169 | N/A | 3 | Urban Planner | 1 | 7 | 9 | 9 | 9 | 8 | 6 | 9 | 9 |
| 83 | 154.239.157.179 | النزهه | 2 | resident | 4 | 9 | 9 | 7 | 7 | 7 | 6 | 9 | 8 |
| 84 | 154.239.236.220 | المعادى | 3 | Civil Engineer | 2 | 6 | 6 | 6 | 7 | 7 | 8 | 7 | 6 |
| 85 | 154.239.96.160 | زهراء المع� | 3 | resident | 4 | 7 | 9 | 6 | 7 | 6 | 6 | 6 | 6 |
| 86 | 154.239.98.60 | الاستثماري | 2 | resident | 4 | 7 | 8 | 7 | 8 | 7 | 8 | 8 | 7 |
| 87 | 156.174.178.67 | الشيخ زايد | 2 | Architect | 1 | 7 | 9 | 9 | 8 | 9 | 9 | 9 | 9 |
| 88 | 156.174.183.155 | المعادي | 1 | resident | 4 | 6 | 8 | 8 | 7 | 8 | 9 | 8 | 7 |
| 89 | 156.178.44.77 | الشيخ زايد | 3 | Realtor | 3 | 6 | 9 | 1 | 5 | 5 | 9 | 9 | 9 |
| 90 | 156.179.38.61 | ٦ أكتوبر | 2 | resident | 4 | 5 | 5 | 7 | 4 | 8 | 7 | 4 | 1 |
| 91 | 156.182.65.234 | زايد | 2 | resident | 4 | 7 | 9 | 7 | 7 | 8 | 8 | 7 | 6 |
| 92 | 156.187.198.81 | Madinty | 1 | RealEstate Investor | 3 | 5 | 9 | 5 | 5 | 5 | 5 | 3 | 3 |
| 93 | 156.187.238.146 | الشروق | 1 | Architect | 1 | 9 | 9 | 7 | 7 | 7 | 7 | 7 | 7 |
| 94 | 156.193.204.175 | الهرم | 2 | Urban Planner | 1 | 9 | 9 | 9 | 9 | 9 | 9 | 9 | 9 |
| 95 | 156.193.82.196 | المريوطية | 2 | resident | 4 | 7 | 8 | 5 | 5 | 7 | 6 | 6 | 5 |
| 96 | 156.200.157.55 | ٦ آكتوبر | 1 | Architect | 1 | 9 | 9 | 8 | 8 | 8 | 9 | 9 | 9 |
| 97 | 156.202.9.94 | القاهره ال� | 2 | Architect | 1 | 6 | 7 | 5 | 5 | 5 | 5 | 5 | 5 |
| 98 | 156.204.154.231 | الرحاب | 1 | Contractor | 2 | 7 | 9 | 4 | 3 | 5 | 5 | 6 | 4 |
| 99 | 156.204.173.95 | شيراتون - ا� | 2 | Architect | 1 | 9 | 9 | 9 | 8 | 9 | 9 | 8 | 9 |
| 100 | 156.204.204.8 | مدينة نصر | 1 | resident | 4 | 9 | 9 | 8 | 8 | 9 | 8 | 7 | 8 |
| 101 | 156.204.241.18 | م.نصر | 3 | resident | 4 | 7 | 9 | 7 | 7 | 7 | 9 | 9 | 7 |
| 102 | 156.204.29.226 | مدينة نصر | 2 | Realtor | 3 | 8 | 9 | 4 | 6 | 5 | 9 | 7 | 4 |
| 103 | 156.204.3.151 | مصر الجديد� | 2 | resident | 4 | 9 | 9 | 9 | 9 | 9 | 9 | 9 | 7 |
| 104 | 156.204.32.53 | N/A | 3 | Resident | 4 | 7 | 9 | 9 | 9 | 9 | 8 | 6 | 8 |
| 105 | 156.204.61.209 | مدينة نصر | 3 | Architect | 1 | 9 | 9 | 9 | 9 | 9 | 9 | 9 | 9 |
| 106 | 156.204.87.96 | مدينه نصر | 2 | resident | 4 | 8 | 9 | 9 | 9 | 9 | 9 | 9 | 9 |
| 107 | 156.204.97.223 | مدينة نصر | 2 | resident | 4 | 9 | 9 | 5 | 5 | 7 | 9 | 5 | 5 |
| 108 | 156.205.217.250 | N/A | 2 | resident | 4 | 9 | 9 | 9 | 7 | 9 | 9 | 9 | 7 |
| 109 | 156.205.234.228 | N/A | 2 | RealEstate Investor | 3 | 7 | 9 | 7 | 8 | 7 | 8 | 8 | 7 |
| 110 | 156.205.252.120 | المنطقة ال� | 2 | resident | 4 | 6 | 8 | 7 | 7 | 6 | 7 | 7 | 6 |
| 111 | 156.205.43.180 | مدينة الشر� | 1 | Contractor | 2 | 2 | 8 | 2 | 3 | 4 | 9 | 9 | 4 |
| 112 | 156.205.80.37 | دار مصر الع | 3 | resident | 4 | 6 | 8 | 7 | 5 | 7 | 9 | 7 | 7 |
| 113 | 156.208.126.6 | الهرم | 1 | Architect | 1 | 8 | 9 | 9 | 8 | 8 | 9 | 7 | 7 |
| 114 | 156.208.167.64 | حلوان و حدا | 2 | resident | 4 | 9 | 8 | 9 | 9 | 9 | 9 | 9 | 9 |
| 115 | 156.208.181.250 | حدائق الاه� | 2 | Realtor | 3 | 8 | 8 | 9 | 7 | 9 | 8 | 8 | 7 |
| 116 | 156.208.241.143 | اكتوبر | 2 | Realtor | 3 | 8 | 8 | 8 | 8 | 8 | 7 | 9 | 7 |
| 117 | 156.208.241.197 | جيزه | 3 | Developer | 3 | 8 | 9 | 9 | 9 | 9 | 9 | 9 | 9 |
| 118 | 156.213.110.76 | التجمع الا� | 2 | resident | 4 | 7 | 8 | 8 | 8 | 8 | 9 | 9 | 7 |
| 119 | 156.213.117.124 | التجمع الأ� | 1 | resident | 4 | 7 | 8 | 8 | 8 | 8 | 8 | 9 | 8 |
| 120 | 156.213.123.156 | التجمع الخ� | 2 | resident | 4 | 9 | 8 | 8 | 9 | 9 | 9 | 9 | 9 |
| 121 | 156.213.141.127 | مدينتي | 1 | Architect | 1 | 9 | 9 | 9 | 8 | 9 | 7 | 7 | 7 |
| 122 | 156.213.147.239 | الشيخ زايد | 1 | resident | 4 | 8 | 7 | 9 | 9 | 9 | 9 | 9 | 8 |
| 123 | 156.213.184.168 | التجمع الخ� | 1 | resident | 4 | 9 | 9 | 6 | 7 | 7 | 7 | 7 | 7 |
| 124 | 156.213.19.136 | القاهرة ال� | 2 | Architect | 1 | 8 | 7 | 7 | 6 | 6 | 7 | 8 | 7 |
| 125 | 156.213.20.28 | القاهرة ال� | 3 | resident | 4 | 9 | 9 | 5 | 9 | 9 | 9 | 9 | 9 |
| 126 | 156.213.243.231 | التجمع الخ� | 2 | resident | 4 | 3 | 6 | 3 | 8 | 7 | 7 | 7 | 5 |
| 127 | 156.213.26.208 | Madinaty | 1 | Developer | 3 | 9 | 8 | 7 | 7 | 7 | 9 | 5 | 5 |
| 128 | 156.213.6.202 | N/A | 1 | Contractor | 2 | 8 | 8 | 8 | 7 | 7 | 8 | 8 | 8 |
| 129 | 156.213.66.168 | القاهرة ال� | 2 | Architect | 1 | 5 | 9 | 7 | 5 | 5 | 6 | 9 | 1 |
| 130 | 156.213.93.125 | التجمع الث� | 3 | resident | 4 | 7 | 9 | 6 | 6 | 9 | 9 | 6 | 7 |
| 131 | 156.213.98.52 | التجمع الخ� | 2 | resident | 4 | 7 | 9 | 8 | 8 | 8 | 9 | 9 | 8 |
| 132 | 156.214.213.173 | N/A | 1 | Architect | 1 | 8 | 8 | 6 | 7 | 7 | 7 | 7 | 8 |
| 133 | 156.214.250.233 | الشيخ زايد | 2 | Architect | 1 | 7 | 9 | 7 | 7 | 7 | 7 | 7 | 7 |
| 134 | 156.215.2.163 | Maadi | 2 | resident | 4 | 9 | 9 | 9 | 8 | 8 | 9 | 9 | 9 |
| 135 | 156.215.27.230 | N/A | 2 | resident | 4 | 7 | 9 | 5 | 3 | 6 | 6 | 4 | 4 |
| 136 | 156.215.55.243 | زهراء المع� | 3 | Architect | 1 | 8 | 9 | 9 | 7 | 7 | 9 | 6 | 3 |
| 137 | 156.219.238.255 | عين شمس | 2 | Urban Planner | 1 | 9 | 9 | 9 | 9 | 9 | 9 | 9 | 9 |
| 138 | 156.223.78.199 | التجمع الخ� | 2 | resident | 4 | 8 | 8 | 9 | 8 | 8 | 9 | 9 | 9 |
| 139 | 188.167.169.42 | مساكن شيرا� | 2 | Architect | 1 | 6 | 9 | 5 | 6 | 8 | 7 | 5 | 8 |
| 140 | 196.128.5.0 | أكتوبر | 1 | Realtor | 3 | 8 | 8 | 6 | 5 | 4 | 8 | 7 | 5 |
| 141 | 196.128.8.117 | الشيخ زايد | 2 | Contractor | 2 | 5 | 9 | 8 | 7 | 7 | 5 | 6 | 6 |
| 142 | 196.132.105.20 | الرحاب | 1 | resident | 4 | 9 | 9 | 8 | 7 | 8 | 8 | 7 | 7 |
| 143 | 196.132.106.233 | القاهره ال� | 2 | Architect | 1 | 9 | 9 | 9 | 9 | 9 | 9 | 9 | 9 |
| 144 | 196.132.106.233 | N/A | 2 | Architect | 1 | 8 | 7 | 7 | 7 | 7 | 7 | 7 | 7 |
| 145 | 196.132.108.19 | التجمع | 1 | Developer | 3 | 9 | 9 | 6 | 6 | 6 | 9 | 9 | 9 |
| 146 | 196.132.130.217 | الدقى | 2 | Contractor | 2 | 8 | 8 | 7 | 7 | 8 | 9 | 7 | 5 |
| 147 | 196.132.136.194 | الرحاب | 1 | resident | 4 | 8 | 9 | 7 | 6 | 8 | 8 | 9 | 8 |
| 148 | 196.132.141.135 | N/A | 3 | Architect | 1 | 6 | 9 | 9 | 9 | 9 | 6 | 8 | 8 |
| 149 | 196.132.200.53 | الشيخ زايد | 1 | Developer | 3 | 4 | 5 | 4 | 6 | 6 | 5 | 7 | 7 |
| 150 | 196.132.36.227 | N/A | 2 | resident | 4 | 8 | 9 | 6 | 9 | 9 | 9 | 9 | 9 |
| 151 | 196.132.68.169 | مدينتي | 2 | Architect | 1 | 3 | 9 | 7 | 5 | 5 | 9 | 9 | 9 |
| 152 | 196.132.7.203 | امتداد الم� | 1 | resident | 4 | 7 | 7 | 7 | 7 | 9 | 8 | 8 | 8 |
| 153 | 196.132.70.209 | N/A | 3 | Civil Engineer | 2 | 8 | 9 | 7 | 6 | 6 | 9 | 8 | 7 |
| 154 | 196.132.70.78 | التجمع الا� | 1 | resident | 4 | 9 | 9 | 8 | 8 | 8 | 8 | 7 | 8 |
| 155 | 196.132.9.29 | ٦ اكتوبر | 2 | Developer | 3 | 6 | 9 | 5 | 5 | 9 | 9 | 8 | 7 |
| 156 | 196.132.96.164 | N/A | 2 | Architect | 1 | 9 | 8 | 6 | 6 | 7 | 6 | 7 | 7 |
| 157 | 196.132.97.249 | N/A | 3 | resident | 4 | 5 | 8 | 7 | 7 | 7 | 9 | 9 | 9 |
| 158 | 196.132.97.99 | التجمع الخ� | 2 | resident | 4 | 9 | 9 | 8 | 8 | 9 | 9 | 9 | 9 |
| 159 | 196.152.15.211 | N/A | 2 | Contractor | 2 | 9 | 9 | 9 | 9 | 9 | 9 | 6 | 6 |
| 160 | 196.152.5.155 | Qalubia | 3 | Architect | 1 | 6 | 8 | 8 | 7 | 8 | 9 | 8 | 8 |
| 161 | 196.152.81.179 | مصر الجديد� | 3 | Architect | 1 | 9 | 9 | 1 | 4 | 7 | 8 | 8 | 6 |
| 162 | 196.154.204.16 | المقطم | 2 | resident | 4 | 8 | 8 | 8 | 8 | 8 | 9 | 8 | 8 |
| 163 | 196.155.200.44 | مدينة ٦ أكت | 3 | RealEstate Investor | 3 | 9 | 9 | 9 | 9 | 9 | 9 | 9 | 9 |
| 164 | 196.155.6.165 | N/A | 2 | Architect | 1 | 6 | 9 | 7 | 6 | 7 | 9 | 9 | 8 |
| 165 | 196.156.130.29 | N/A | 1 | Developer | 3 | 8 | 7 | 4 | 3 | 4 | 4 | 4 | 4 |
| 166 | 196.156.18.187 | المنصورية | 1 | Developer | 3 | 5 | 6 | 6 | 1 | 5 | 6 | 7 | 4 |
| 167 | 196.156.18.83 | N/A | 2 | Civil Engineer | 2 | 8 | 8 | 8 | 8 | 8 | 8 | 9 | 8 |
| 168 | 196.156.4.190 | الشيخ زايد | 1 | resident | 4 | 8 | 9 | 7 | 7 | 8 | 9 | 8 | 8 |
| 169 | 196.156.8.172 | اكتوبر | 3 | resident | 4 | 5 | 9 | 5 | 5 | 5 | 5 | 5 | 6 |
| 170 | 196.157.106.253 | مدينة نصر | 2 | Contractor | 2 | 8 | 8 | 8 | 8 | 8 | 8 | 9 | 7 |
| 171 | 196.157.14.26 | الشيخ زايد | 1 | RealEstate Investor | 3 | 8 | 8 | 5 | 3 | 3 | 8 | 3 | 5 |
| 172 | 196.157.3.171 | مدينة ١٥ ما | 3 | Developer | 3 | 7 | 7 | 6 | 7 | 8 | 9 | 8 | 8 |
| 173 | 196.157.32.59 | المعادي | 2 | resident | 4 | 5 | 9 | 9 | 9 | 9 | 9 | 5 | 5 |
| 174 | 196.158.131.236 | مدينة نصر | 2 | Urban Planner | 1 | 6 | 8 | 5 | 6 | 6 | 4 | 7 | 6 |
| 175 | 196.158.133.65 | N/A | 4 | Civil Engineer | 2 | 6 | 7 | 5 | 5 | 5 | 5 | 4 | 6 |
| 176 | 196.158.195.206 | مدينة نصر | 3 | Architect | 1 | 9 | 5 | 5 | 5 | 9 | 6 | 6 | 4 |
| 177 | 196.158.199.142 | التجمع الخ� | 1 | resident | 4 | 8 | 8 | 8 | 8 | 8 | 9 | 9 | 5 |
| 178 | 196.159.7.190 | القاهرة ال� | 1 | Realtor | 3 | 9 | 9 | 8 | 8 | 8 | 7 | 7 | 5 |
| 179 | 196.218.12.24 | el zatoun | 3 | Architect | 1 | 9 | 9 | 9 | 8 | 8 | 9 | 8 | 8 |
| 180 | 196.219.221.178 | N/A | 3 | resident | 4 | 7 | 9 | 7 | 6 | 6 | 9 | 7 | 8 |
| 181 | 196.221.102.147 | مدينة نصر | 1 | Developer | 3 | 9 | 9 | 9 | 9 | 9 | 9 | 9 | 9 |
| 182 | 197.120.135.156 | New Cairo | 2 | Developer | 3 | 9 | 6 | 7 | 7 | 8 | 9 | 7 | 2 |
| 183 | 197.133.79.70 | مدينة نصر | 2 | resident | 4 | 9 | 7 | 8 | 9 | 9 | 9 | 9 | 8 |
| 184 | 197.135.58.247 | N/A | 2 | Architect | 1 | 9 | 9 | 9 | 9 | 9 | 9 | 9 | 9 |
| 185 | 197.196.207.230 | مدينة نصر | 2 | Urban Planner | 1 | 7 | 8 | 9 | 7 | 8 | 9 | 9 | 8 |
| 186 | 197.246.19.227 | المعادي | 2 | resident | 4 |  | 9 | 5 | 5 | 9 | 9 | 9 | 5 |
| 187 | 197.37.114.154 | الزيتون | 3 | resident | 4 | 7 | 9 | 8 | 9 | 7 | 9 | 9 | 9 |
| 188 | 197.37.128.224 | مصرالجديدة | 2 | resident | 4 | 9 | 9 | 9 | 9 | 9 | 9 | 9 | 9 |
| 189 | 197.37.139.0 | النزهة | 2 | Architect | 1 | 6 | 8 | 6 | 6 | 6 | 7 | 6 | 6 |
| 190 | 197.37.146.255 | جمال عبد ال | 2 | Resident | 4 | 5 | 5 | 5 | 5 | 5 | 5 | 5 | 5 |
| 191 | 197.37.223.28 | جسر السويس | 2 | resident | 4 | 9 | 9 | 8 | 8 | 8 | 9 | 9 | 8 |
| 192 | 197.37.226.198 | مصر الجديد� | 3 | Architect | 1 | 8 | 9 | 6 | 6 | 7 | 8 | 8 | 8 |
| 193 | 197.39.220.151 | N/A | 3 | Civil Engineer | 2 | 7 | 9 | 8 | 7 | 8 | 8 | 9 | 8 |
| 194 | 197.43.246.39 | شيراتون | 1 | RealEstate Investor | 3 | 9 | 9 | 9 | 9 | 9 | 9 | 9 | 9 |
| 195 | 197.46.19.190 | مدينه نصر | 2 | resident | 4 | 7 | 9 | 5 | 6 | 8 | 9 | 9 | 9 |
| 196 | 197.46.97.239 | مدينة نصر | 3 | resident | 4 | 8 | 9 | 9 | 9 | 9 | 9 | 9 | 9 |
| 197 | 197.47.114.177 | التجمع الخ� | 2 | resident | 4 | 8 | 9 | 8 | 9 | 9 | 9 | 9 | 4 |
| 198 | 197.47.117.37 | التجمع | 1 | resident | 4 | 8 | 8 | 7 | 6 | 6 | 7 | 7 | 6 |
| 199 | 197.47.132.173 | التجمع الخ� | 2 | Architect | 1 | 7 | 9 | 8 | 7 | 8 | 9 | 8 | 5 |
| 200 | 197.47.132.21 | الرحاب | 2 | resident | 4 | 6 | 8 | 6 | 8 | 8 | 9 | 9 | 8 |
| 201 | 197.47.151.30 | التجمع الأ� | 2 | Architect | 1 | 8 | 8 | 7 | 7 | 7 | 8 | 8 | 7 |
| 202 | 197.47.153.220 | التجمع الأ� | 2 | resident | 4 | 8 | 9 | 9 | 9 | 9 | 9 | 9 | 8 |
| 203 | 197.47.162.179 | النرجس | 2 | resident | 4 | 8 | 8 | 8 | 8 | 8 | 8 | 8 | 8 |
| 204 | 197.47.17.8 | التجمع | 2 | Architect | 1 | 8 | 7 | 7 | 9 | 9 | 8 | 8 | 9 |
| 205 | 197.47.19.162 | الرحاب | 1 | Developer | 3 | 8 | 9 | 7 | 7 | 8 | 8 | 9 | 8 |
| 206 | 197.47.205.153 | N/A | 2 | Developer | 3 | 7 | 7 | 6 | 7 | 7 | 8 | 9 | 7 |
| 207 | 197.47.224.50 | التجمع الخ� | 2 | resident | 4 | 9 | 9 | 9 | 9 | 9 | 9 | 9 | 9 |
| 208 | 197.47.236.111 | N/A | 3 | resident | 4 | 6 | 9 | 8 | 8 | 8 | 9 | 9 | 7 |
| 209 | 197.47.250.19 | التجمع | 1 | resident | 4 | 8 | 8 | 7 | 4 | 7 | 9 | 9 | 9 |
| 210 | 197.47.30.32 | New cairo | 3 | resident | 4 | 8 | 9 | 6 | 6 | 8 | 8 | 8 | 8 |
| 211 | 197.47.37.174 | القاهرة ال� | 1 | RealEstate Investor | 3 | 6 | 7 | 6 | 5 | 7 | 7 | 8 | 6 |
| 212 | 197.47.43.63 | شرق الأكاد� | 1 | Civil Engineer | 2 | 8 | 9 | 9 | 8 | 8 | 9 | 9 | 8 |
| 213 | 197.47.64.135 | التجمع الخ� | 2 | resident | 4 | 9 | 9 | 9 | 7 | 8 | 8 | 9 | 9 |
| 214 | 197.47.87.73 | الرحاب | 2 | resident | 4 | 8 | 9 | 9 | 9 | 9 | 9 | 9 | 7 |
| 215 | 197.48.10.120 | N/A | 1 | Contractor | 2 | 9 | 9 | 9 | 9 | 9 | 9 | 9 | 9 |
| 216 | 197.50.105.175 | العبور الح� | 1 | resident | 4 | 4 | 8 | 5 | 3 | 6 | 8 | 8 | 6 |
| 217 | 197.52.195.72 | المقطم | 3 | Architect | 1 | 7 | 9 | 7 | 6 | 8 | 8 | 9 | 8 |
| 218 | 197.52.32.227 | حلوان | 2 | Architect | 1 | 5 | 8 | 6 | 5 | 7 | 7 | 6 | 7 |
| 219 | 197.53.235.7 | ٦ أكتوبر | 3 | Architect | 1 | 5 | 9 | 4 | 3 | 5 | 6 | 3 | 9 |
| 220 | 197.57.147.73 | مدينة نصر | 2 | resident | 4 | 7 | 9 | 7 | 7 | 7 | 9 | 9 | 9 |
| 221 | 197.57.150.69 | مدينه نصر | 3 | Architect | 1 | 7 | 9 | 7 | 5 | 6 | 8 | 7 | 7 |
| 222 | 197.62.15.131 | N/A | 3 | Architect | 1 | 8 | 9 | 7 | 8 | 8 | 9 | 8 | 9 |
| 223 | 203.220.128.60 | N/A | 2 | resident | 4 | 9 | 9 | 7 | 7 | 7 | 9 | 9 | 7 |
| 224 | 213.212.214.51 | معادي | 2 | RealEstate Investor | 3 | 9 | 9 | 9 | 8 | 7 | 8 | 7 | 7 |
| 225 | 217.55.121.243 | الشيخ زايد | 1 | resident | 4 | 9 | 9 | 7 | 7 | 7 | 9 | 9 | 8 |
| 226 | 217.55.144.101 | شيراتون | 1 | resident | 4 | 8 | 9 | 9 | 9 | 7 | 5 | 5 | 8 |
| 227 | 217.55.71.221 | المعادي | 2 | resident | 4 | 4 | 9 | 8 | 8 | 8 | 8 | 9 | 6 |
| 228 | 31.167.88.26 | مدينتي الق� | 2 | resident | 4 | 8 | 8 | 7 | 7 | 8 | 9 | 8 | 7 |
| 229 | 31.219.123.119 | N/A | 3 | Architect | 1 | 8 | 9 | 8 | 8 | 7 | 6 | 9 | 6 |
| 230 | 31.219.147.69 | مدينة الشر� | 1 | Architect | 1 | 8 | 7 | 7 | 7 | 7 | 5 | 8 | 8 |
| 231 | 37.201.224.17 | مدينتي | 2 | resident | 4 | 4 | 9 | 8 | 4 | 6 | 8 | 3 | 3 |
| 232 | 41.129.23.54 | New cairo | 2 | resident | 4 | 4 | 8 | 9 | 1 | 1 | 2 | 1 | 1 |
| 233 | 41.129.89.31 | مدينه العب� | 3 | Architect | 1 | 7 | 9 | 8 | 6 | 8 | 9 | 9 | 8 |
| 234 | 41.199.107.234 | المعادي | 2 | resident | 4 | 6 | 7 | 6 | 7 | 7 | 8 | 7 | 6 |
| 235 | 41.199.181.152 | مدينة نصر | 3 | resident | 4 | 9 | 9 | 7 | 7 | 7 | 9 | 8 | 5 |
| 236 | 41.218.182.216 | مدينة نصر | 3 | resident | 4 | 7 | 9 | 6 | 5 | 6 | 8 | 7 | 4 |
| 237 | 41.232.164.106 | المقطم | 3 | Architect | 1 | 9 | 9 | 4 | 7 | 7 | 9 | 9 | 9 |
| 238 | 41.234.10.6 | المقطم | 2 | Contractor | 2 | 7 | 6 | 7 | 7 | 7 | 7 | 7 | 7 |
| 239 | 41.234.71.3 | ٦ اكتوبر | 2 | Contractor | 2 | 8 | 9 | 9 | 9 | 8 | 9 | 7 | 8 |
| 240 | 41.236.137.188 | N/A | 3 | Realtor | 3 | 7 | 9 | 4 | 4 | 5 | 9 | 9 | 9 |
| 241 | 41.236.26.252 | النزهة | 1 | Civil Engineer | 2 | 7 | 9 | 5 | 5 | 5 | 5 | 5 | 5 |
| 242 | 41.236.58.188 | N/A | 1 | resident | 4 | 8 | 8 | 4 | 5 | 6 | 9 | 9 | 6 |
| 243 | 41.237.128.110 | N/A | 2 | resident | 4 | 8 | 9 | 7 | 6 | 7 | 9 | 7 | 7 |
| 244 | 41.237.177.19 | N/A | 2 | Architect | 1 | 9 | 9 | 9 | 9 | 9 | 9 | 9 | 9 |
| 245 | 41.237.197.74 | Qalyubia | 3 | Contractor | 2 | 7 | 9 | 7 | 7 | 7 | 8 | 8 | 8 |
| 246 | 41.239.173.228 | المعادي | 2 | Architect | 1 | 8 | 9 | 5 | 5 | 6 | 8 | 7 | 7 |
| 247 | 41.33.6.100 | الشروق | 3 | resident | 4 | 8 | 9 | 8 | 8 | 8 | 9 | 9 | 8 |
| 248 | 41.33.67.36 | حي الرحاب | 1 | Architect | 1 | 5 | 9 | 9 | 9 | 9 | 6 | 9 | 6 |
| 249 | 41.34.51.27 | N/A | 2 | Civil Engineer | 2 | 1 | 9 | 9 | 1 | 1 | 9 | 9 | 9 |
| 250 | 41.35.114.232 | مدينه الشر� | 2 | resident | 4 | 7 | 9 | 8 | 8 | 9 | 9 | 9 | 9 |
| 251 | 41.35.115.177 | مدينة الشر� | 2 | resident | 4 | 9 | 9 | 9 | 9 | 9 | 9 | 9 | 9 |
| 252 | 41.35.243.1 | مساكن شيرا� | 1 | resident | 4 | 9 | 9 | 7 | 4 | 5 | 5 | 4 | 5 |
| 253 | 41.40.213.147 | N/A | 1 | Developer | 3 | 8 | 9 | 7 | 7 | 7 | 8 | 8 | 8 |
| 254 | 41.42.170.133 | زهراء مدين� | 3 | Architect | 1 | 7 | 9 | 8 | 7 | 6 | 8 | 8 | 6 |
| 255 | 41.42.209.17 | العبور | 1 | Architect | 1 | 6 | 9 | 9 | 6 | 6 | 3 | 9 | 9 |
| 256 | 41.42.70.208 | Elshikh zayad | 1 | Architect | 1 | 9 | 9 | 9 | 9 | 9 | 9 | 9 | 9 |
| 257 | 41.43.50.32 | N/A | 2 | Architect | 1 | 7 | 8 | 7 | 7 | 8 | 7 | 7 | 8 |
| 258 | 41.43.55.238 | العبور | 1 | resident | 4 | 9 | 9 | 9 | 9 | 9 | 9 | 9 | 9 |
| 259 | 41.43.85.60 | مصر الجديد� | 2 | resident | 4 | 7 | 7 | 6 | 7 | 8 | 8 | 8 | 1 |
| 260 | 41.44.40.16 | النزهه الج� | 3 | Architect | 1 | 9 | 9 | 9 | 9 | 5 | 9 | 9 | 9 |
| 261 | 41.44.53.248 | بني سويف | 1 | Architect | 1 | 9 |  | 8 | 7 | 8 | 9 | 9 | 9 |
| 262 | 41.45.107.175 | مدينة نصر | 2 | Architect | 1 | 5 | 7 | 5 | 6 | 4 | 7 | 7 | 4 |
| 263 | 41.45.238.202 | التجمع الخ� | 2 | resident | 4 | 9 | 9 | 9 | 9 | 9 | 9 | 8 | 8 |
| 264 | 41.46.136.125 | المعادي | 2 | resident | 4 | 9 | 9 | 9 | 9 | 9 | 9 | 9 | 9 |
| 265 | 41.46.167.78 | الجيزة | 3 | resident | 4 | 7 | 8 | 6 | 6 | 6 | 6 | 6 | 6 |
| 266 | 41.47.20.66 | المهندسين | 3 | Architect | 1 | 5 | 8 | 9 | 6 | 9 | 9 | 9 | 8 |
| 267 | 41.65.176.39 | الشيخ زايد | 1 | Architect | 1 | 7 | 7 | 7 | 8 | 7 | 9 | 7 | 8 |
| 268 | 41.65.176.42 | المهندسين | 2 | Interior Designer | 1 | 7 | 7 | 5 | 5 | 5 | 7 | 5 | 9 |
| 269 | 41.68.137.241 | التجمع الا� | 2 | resident | 4 | 7 | 9 | 7 | 7 | 9 | 9 | 9 | 9 |
| 270 | 41.69.208.5 | مصر الجديد� | 1 | Architect | 1 | 8 | 9 | 9 | 7 | 9 | 9 | 9 | 9 |
| 271 | 41.69.68.206 | N/A | 2 | Developer | 3 | 7 | 8 | 4 | 7 | 9 | 9 | 9 | 8 |
| 272 | 41.69.91.148 | الهرم | 3 | Contractor | 2 | 9 | 9 | 7 | 7 | 9 | 9 | 9 | 8 |
| 273 | 43.132.162.28 | N/A | 3 | resident | 4 | 7 | 9 | 9 | 9 | 9 | 9 | 9 | 9 |
| 274 | 45.100.108.42 | الشيخ زايد | 1 | resident | 4 | 3 | 6 | 3 | 3 | 8 | 6 | 8 | 5 |
| 275 | 45.105.14.184 | ٦ اكتوبر | 1 | RealEstate Investor | 3 | 8 | 9 | 7 | 7 | 7 | 7 | 9 | 8 |
| 276 | 45.135.187.77 | مدينتي | 1 | Architect | 1 | 7 | 9 | 7 | 7 | 7 | 7 | 7 | 7 |
| 277 | 45.240.120.140 | مدينة ٦ اكت | 1 | RealEstate Investor | 3 | 8 | 8 | 8 | 8 | 8 | 9 | 9 | 8 |
| 278 | 45.240.185.254 | التجمع الخ� | 2 | Architect | 1 | 4 | 7 | 7 | 7 | 7 | 7 | 9 | 7 |
| 279 | 45.240.24.168 | N/A | 2 | resident | 4 | 7 | 9 | 6 | 5 | 6 | 8 | 8 | 8 |
| 280 | 45.240.241.96 | الرحاب | 1 | resident | 4 | 7 | 9 | 9 | 9 | 9 | 9 | 9 | 9 |
| 281 | 45.240.243.248 | N/A | 1 | resident | 4 | 8 | 8 | 6 | 6 | 6 | 8 | 8 | 8 |
| 282 | 45.243.222.135 | التجمع | 2 | resident | 4 | 9 | 9 | 7 | 9 | 9 | 6 | 9 | 8 |
| 283 | 45.247.43.10 | القاهرة ال� | 1 | resident | 4 | 8 | 8 | 8 | 7 | 7 | 7 | 7 | 7 |
| 284 | 45.247.64.204 | مدينة نصر | 2 | resident | 4 | 5 | 8 | 8 | 6 | 7 | 9 | 7 | 5 |
| 285 | 45.62.219.161 | N/A | 1 | resident | 4 | 7 | 9 | 8 | 6 | 9 | 9 | 9 | 8 |
| 286 | 45.96.193.240 | الهرم | 4 | Architect | 1 | 5 | 9 | 5 | 4 | 5 | 9 | 5 | 9 |
| 287 | 45.97.112.69 | شبرا مصر | 3 | Architect | 1 | 8 | 7 | 6 | 5 | 5 | 9 | 9 | 8 |
| 288 | 45.98.219.252 | حدائق الاه� | 3 | RealEstate Investor | 3 | 5 | 9 | 6 | 8 | 6 | 9 | 7 | 7 |
| 289 | 46.142.193.10 | N/A | 1 | Developer | 3 | 6 | 9 | 4 | 4 | 7 | 7 | 8 | 5 |
| 290 | 5.110.175.237 | مدينة 6 اكت� | 2 | Urban Planner | 1 | 7 | 8 | 8 | 6 | 8 | 8 | 8 | 4 |
| 291 | 5.110.19.40 | N/A | 3 | Architect | 1 | 6 | 8 | 6 | 6 | 7 | 6 | 7 | 6 |
| 292 | 5.194.174.19 | التجمع الخ� | 1 | resident | 4 | 6 | 7 | 7 | 7 | 7 | 7 | 9 | 7 |
| 293 | 51.211.220.82 | التجمع الخ� | 1 | resident | 4 | 7 | 8 | 8 | 7 | 1 | 8 | 8 | 8 |
| 294 | 51.252.45.100 | المقطم | 2 | resident | 4 | 7 | 7 | 7 | 7 | 8 | 7 | 7 | 5 |
| 295 | 51.39.122.79 | حدائق القب� | 2 | Architect | 1 | 7 | 9 | 7 | 7 | 8 | 7 | 9 | 9 |
| 296 | 51.39.188.169 | مدينه الشر� | 1 | Interior Designer | 1 | 7 | 9 | 9 | 8 | 8 | 9 | 8 | 8 |
| 297 | 51.39.198.91 | Zayed | 2 | Architect | 1 | 6 | 6 | 6 | 5 | 3 | 9 | 9 | 9 |
| 298 | 68.82.59.145 | مدينه نصر | 1 | resident | 4 | 8 | 7 | 7 | 5 | 7 | 9 | 8 | 7 |
| 299 | 73.76.50.173 | المعادي | 1 | resident | 4 | 8 | 8 | 2 | 2 | 2 | 8 | 1 | 1 |
| 300 | 76.220.72.135 | التجمع الخ� | 2 | Contractor | 2 | 6 | 9 | 7 | 7 | 6 | 7 | 8 | 7 |
| 301 | 78.100.237.16 | المعادي | 2 | Interior Designer | 1 | 7 | 9 | 7 | 8 | 7 | 9 | 9 | 9 |
| 302 | 80.76.166.90 | مصر الجديد� | 2 | resident | 4 | 5 | 9 | 5 | 5 | 5 | 5 | 5 | 5 |
| 303 | 82.129.239.50 | البارون سي� | 2 | resident | 4 | 7 | 8 | 6 | 7 | 7 | 9 | 7 | 6 |
| 304 | 86.60.55.27 | الاسماعيلي | 2 | Developer | 3 | 7 | 9 | 7 | 7 | 7 | 8 | 8 | 7 |
| 305 | 86.97.181.29 | مدينتي | 2 | Civil Engineer | 2 | 8 | 8 | 4 | 8 | 8 | 9 | 9 | 9 |
| 306 | 86.97.53.128 | التجمع الخ� | 1 | resident | 4 | 7 | 9 | 5 | 3 | 5 | 5 | 9 | 9 |
| 307 | 89.1.74.190 | Nasr City | 3 | resident | 4 | 7 | 7 | 7 | 7 | 7 | 7 | 7 | 7 |
| 308 | 90.252.186.7 | مدينة الشر� | 1 | Civil Engineer | 2 |  | 9 | 9 | 9 | 9 | 9 | 9 | 9 |
| 309 | 91.73.20.125 | N/A | 1 | resident | 4 | 7 | 9 | 8 | 8 | 8 | 8 | 8 | 8 |
| 310 | 92.8.82.70 | المعادي | 2 | Architect | 1 | 7 | 9 | 9 | 5 | 8 | 7 | 5 | 5 |
| 311 | 92.98.98.162 | اكتوبر | 2 | Architect | 1 | 6 | 8 | 6 | 7 | 7 | 7 | 8 | 8 |
| 312 | 94.201.98.107 | مدينه نصر | 2 | resident | 4 | 9 | 9 | 9 | 9 | 9 | 9 | 9 | 9 |
| 313 | 94.203.18.209 | التجمع الخ� | 2 | Architect | 1 | 9 | 7 | 9 | 6 | 9 | 9 | 8 | 9 |
| 314 | 94.205.32.102 | التجمع الخ� | 1 | Urban Planner | 1 | 3 | 7 | 3 | 3 | 7 | 9 | 3 | 4 |
| 315 | 94.207.52.127 | عين شمس | 3 | resident | 4 | 9 | 9 | 9 | 7 | 7 | 8 | 7 | 8 |
